# Neurofeedback and attention modulate somatosensory alpha oscillations but not pain perception

**DOI:** 10.1371/journal.pbio.3002972

**Published:** 2025-01-23

**Authors:** Vanessa D. Hohn, Laura Tiemann, Felix S. Bott, Elisabeth S. May, Clara Fritzen, Moritz M. Nickel, Cristina Gil Ávila, Markus Ploner

**Affiliations:** 1 Department of Neurology, School of Medicine and Health, Technical University of Munich (TUM), Munich, Germany; 2 TUM-Neuroimaging Center, School of Medicine and Health, TUM, Munich, Germany; 3 Center for Interdisciplinary Pain Medicine, School of Medicine and Health, TUM, Munich, Germany; Universidade Federal de Santa Catarina, BRAZIL

## Abstract

Pain is closely linked to alpha oscillations (8 < 13 Hz) which are thought to represent a supra-modal, top-down mediated gating mechanism that shapes sensory processing. Consequently, alpha oscillations might also shape the cerebral processing of nociceptive input and eventually the perception of pain. To test this mechanistic hypothesis, we designed a sham-controlled and double-blind electroencephalography (EEG)-based neurofeedback study. In a short-term neurofeedback training protocol, healthy participants learned to up- and down-regulate somatosensory alpha oscillations using attention. Subsequently, we investigated how this manipulation impacts experimental pain applied during neurofeedback. Using Bayesian statistics and mediation analysis, we aimed to test whether alpha oscillations mediate attention effects on pain perception. The results showed that attention and neurofeedback successfully up- and down-regulated the asymmetry of somatosensory alpha oscillations. However, attention and neurofeedback did not modulate pain ratings or related brain responses. Accordingly, somatosensory alpha oscillations did not mediate attention effects on pain perception. Thus, our results challenge the hypothesis that somatosensory alpha oscillations shape pain perception. A causal relationship between alpha oscillations and pain perception might not exist or be more complex than hypothesized.

**Trial registration:** Following Stage 1 acceptance, the study was registered at ClinicalTrials.gov NCT05570695.

## Abstract 2

Alpha oscillations shape sensory processing, but do they also modulate the perception of pain? This pre-registered study found no evidence for a causal relationship between alpha oscillations and pain perception.

## Introduction

Pain is a vital phenomenon that emerges from complex patterns of brain activity. These patterns extend across a broad network of brain areas, which generate neuronal oscillations at different frequencies, ranging from infra-slow oscillations below 0.1 Hz to high-frequency oscillations up to 100 Hz [1,2]. Among these oscillations, alpha oscillations at frequencies between 8 to 13 Hz have been particularly related to pain. Specifically, studies have shown that the power of alpha oscillations after and even before a noxious event inversely relates to the intensity of pain [3–7]. These correlative findings match current theories, which propose that alpha oscillations gate sensory information processing in the human brain [8–10]. While lower alpha power in task-relevant, sensory regions is thought to facilitate processing of relevant sensory input, higher alpha power in task-irrelevant brain areas is thought to inhibit processing of irrelevant input. Attention represents one of the drivers that activate this alpha-based gating mechanism [11,12] and has also been shown to influence pain perception [13] as well as associated brain responses [14,15]. Correspondingly, attention-related alpha oscillations in somatosensory brain areas might serve the gating of nociceptive information in the processing of pain [14,16] and might therefore represent an ideal access point to the brain network underlying pain perception. If so, modulations of these oscillations should result in modulations of pain. Specifically, lower alpha power in relevant, contralateral sensory areas should facilitate the processing of nociceptive input leading to more pain, whereas higher alpha power in these areas should inhibit the processing of nociceptive input leading to less pain. However, causal evidence for a mechanistic relationship between attention, alpha oscillations, and pain is lacking so far.

Neurofeedback is an increasingly popular, noninvasive method used to modulate brain activity and enhance cognitive functions such as attention in human participants [17,18]. During neurofeedback, participants learn to purposefully regulate their brain activity by obtaining real-time feedback regarding their brain activity. Subsequently, the influence of this regulation on behavior can be investigated. The appeal of such neurofeedback approaches is twofold [19]. First, modulating brain activity and observing which effects this has on behavior allows researchers to identify causal relationships between brain and behavior. Second, if causal brain–behavior relationships exist, neurofeedback can be used to modulate and treat abnormal perception and behavior in neuropsychiatric disorders. The latter is particularly appealing when broadly available and cost-effective neurofeedback approaches based on electroencephalography (EEG) are used [20]. Consequently, a surge of EEG-based neurofeedback approaches is currently observed, and their results and perspectives are lively and controversially discussed [21–26].

Despite the conceptual plausibility of alpha neurofeedback in the context of pain [27], its impact on clinical and experimental pain remains unclear. While some studies report significant pain reductions, others did not find improvements [20,28]. This inconsistency might be due to the particular methodological challenges of neurofeedback studies. One major challenge is to directly link modulations of brain activity to modulations of perception, e.g., by showing concomitant changes of brain activity and perception during neurofeedback instead of relying on pre-post neurofeedback changes only. Another crucial challenge is to define control conditions which effectively separate neurofeedback-specific effects from nonspecific effects such as time and placebo effects [20,28]. So far, most pain-related studies have not met these challenges rendering the specificity and significance of previous findings unclear [20,28].

With these specific challenges in mind, we designed an EEG-based neurofeedback study to investigate whether modulating attention-related alpha oscillations in somatosensory regions can modulate pain perception and underlying brain responses. In a within-subjects design, we implemented a short-term neurofeedback training targeting attention-related alpha oscillations and investigated its impact on experimental pain. To establish a direct link between alpha oscillations and pain, we applied brief noxious laser stimuli to the left hand during the neurofeedback training and related modulations of alpha oscillations to modulations of pain perception on a trial-by-trial basis [29]. To enhance the specificity of our training, we trained participants to regulate the asymmetry of somatosensory alpha oscillations between hemispheres instead of focusing on alpha oscillations in one hemisphere only. Alpha asymmetry has been successfully modulated in recent neurofeedback studies [30,31] and effectively controls for muscle activity and arousal effects which typically yield local or global changes of brain activity but not changes in the asymmetry of oscillations. In addition, alpha asymmetry can be modulated by either up- and down-regulating alpha oscillations in a certain hemisphere or both, making (1) it less prone to time effects; and (2) its up- and down-regulation comparable in difficulty. To further control for nonspecific effects [17] and achieve double-blinding, we implemented a sham-controlled bidirectional regulation design [32] during which alpha asymmetry is regulated in opposite directions. In a first verum condition, participants were instructed to focus attention on their right hand and the up-regulation of alpha oscillations in the right (contralateral to noxious stimulation) hemisphere relative to alpha oscillations in the left (ipsilateral to noxious stimulation) hemisphere was incentivized through neurofeedback (attention right training, ART_NF_). In a second verum condition, participants were instructed to focus attention on their left hand and the down-regulation of right relative to left alpha oscillations was incentivized (attention left training, ALT_NF_). We hypothesized that, if a direct link between attention-related alpha oscillations and pain exists, this bidirectional regulation should result in opposite behavioral effects [32], which should be less pronounced in the 2 sham conditions with yoked feedback (ART_sham_ and ALT_sham_).

**Fig 1:**
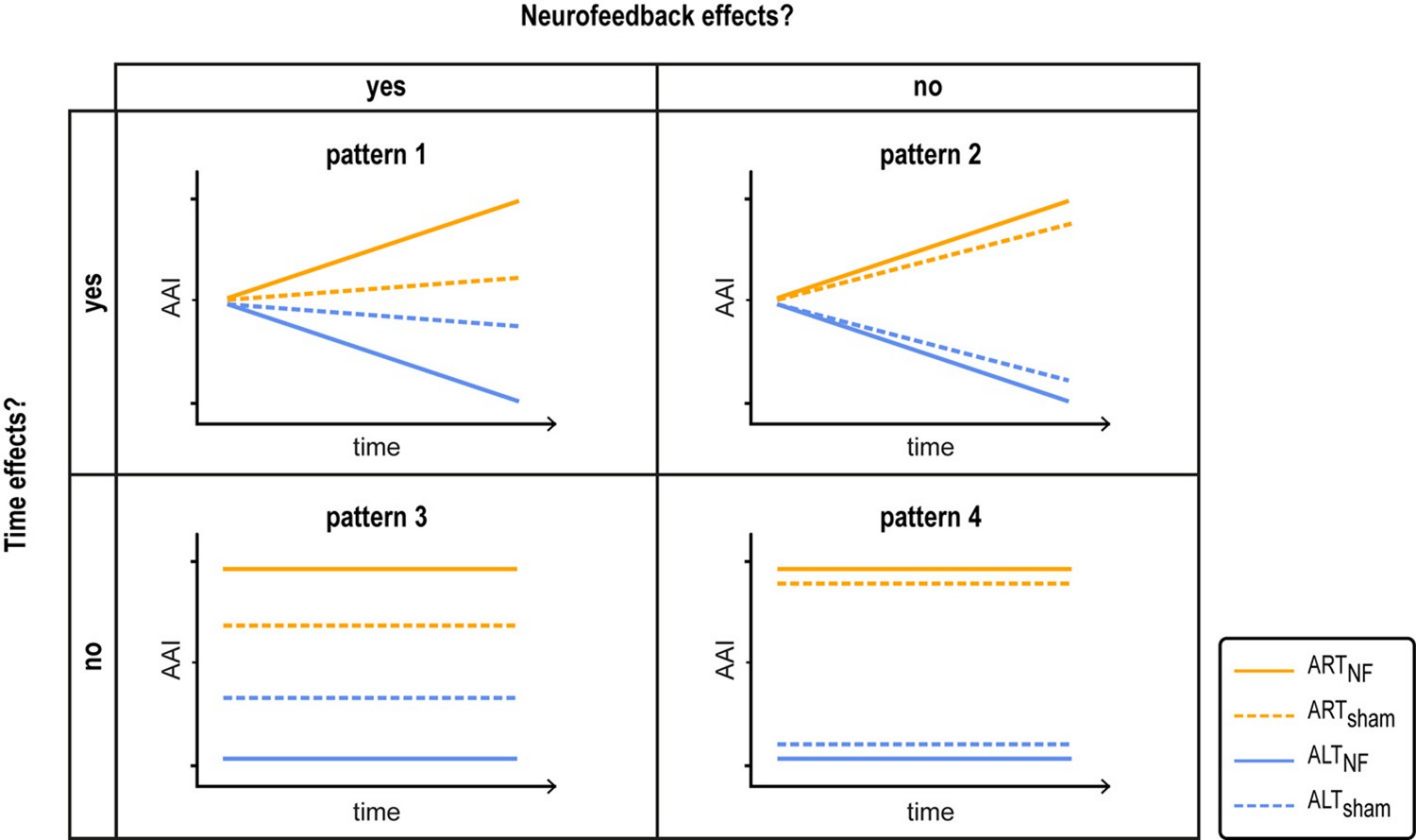
Predicted result patterns characterizing attention effects on alpha asymmetry. Four different result patterns characterizing attention effects on alpha asymmetry indices (AAI, quantified as the normalized difference of alpha oscillations over right minus left somatosensory brain areas) were investigated. Attention effects on AAIs manifest as difference between ART and ALT (orange and blue lines) and can be complemented by neurofeedback and time effects. Neurofeedback effects quantify differences between verum and sham neurofeedback conditions (solid and dashed lines). Time effects quantify differences between the first and second half of the data. For pain ratings and underlying brain responses, a reversal of attention effects, i.e., a down-regulation of pain during ART and an up-regulation during ALT, would be expected due to the inverse relationship between alpha oscillations and pain. Note that the figure entails a schematic representation of the change of AAI values across time/trials and does not reflect expected absolute values. AAI, alpha asymmetry index; ALT_NF/sham_, verum/sham attention left training; ART_NF/sham_, verum/sham attention right training.

To test whether a mechanistic relationship between attention, alpha oscillations, and pain exists, we compared the findings to 4 predicted result patterns capturing different combinations of attention, neurofeedback, and time effects (Fig 1) on alpha oscillations (hypothesis 1) and on pain perception and underlying brain responses (hypothesis 2). In addition, we performed a multilevel mediation analysis based on single trial data to test whether changes in attention, alpha oscillations, and pain perception can be integrated into a single, mechanistic model (hypothesis 3). All analyses relied on Bayesian hypothesis testing, allowing us to quantify evidence in favor as well as against our hypotheses [33].

Hypothesis 1: Alpha oscillations are up- and down-regulated. Based on the literature and pilot data (Fig G in S1 Text), we expected that lateralized attention would lead to an up-regulation of alpha asymmetry in ART conditions and a down-regulation in ALT conditions. Moreover, we expected that these attention effects would be enhanced through verum compared to sham neurofeedback and increase over time (H1.1–3, Fig 1, pattern 1).
Hypothesis 2: The perception of pain and underlying brain responses are up- and down-regulated. Assuming that attention-related alpha oscillations are causally involved in the emergence of pain, we also expected pain ratings to be up- and down-regulated. During ART, the brain should be optimally tuned for the processing of stimuli from the right body side, thus leading to lower pain ratings in response to the applied left-sided pain stimuli. During ALT, the brain should be optimally tuned for the processing of stimuli from the left side of the body which should result in higher pain ratings. Hence, pain ratings should be lower during ART compared to ALT conditions. As for alpha asymmetry, we expected this attention effect to be enhanced through verum compared to sham neurofeedback (H2.1–2, Fig 1, pattern 1 or 3). Time effects were not analyzed due to confounding effects of habituation or sensitization. To examine whether attention affects neural pain processing in a similar fashion, corresponding hypotheses were tested for brain responses to noxious stimuli [34] (H2.3–4, Fig 1, pattern 1 or 3).
Hypothesis 3: Alpha oscillations partially mediate attention effects on pain perception. To test this hypothesis, we employed multilevel mediation analysis. Mediation analysis not only assesses the effects of an independent variable (X) on a dependent variable (Y), but quantifies to which extent a third variable termed mediator (M) contributes to these effects [35]. Using this model, we expected that alpha oscillations (M) would partially mediate the relationship between ART/ALT neurofeedback conditions (X) and pain ratings (Y), and that this mediation effect would be more pronounced for the verum than for the sham neurofeedback (moderator) (H3).

In summary, we performed a rigorous, double-blind and fully transparent neurofeedback study with state-of-the-art control for nonspecific effects, which is being increasingly called for by the neurofeedback community in the field of pain research [20,28] and beyond [17]. The study was analyzed using contemporary Bayesian hypothesis testing and multilevel mediation analysis. As a result, the study provides novel mechanistic insights into the role of attention-related alpha oscillations for pain. Beyond, it is essential for informing the development of urgently needed noninvasive treatment approaches for chronic pain.

## Methods

### Ethics

The study protocol was approved by the local Ethics Committee of the Medical Faculty of the Technical University of Munich (2020–780_1-S-SR). Following Stage 1 acceptance, the study was registered at ClinicalTrials.gov (https://clinicaltrials.gov/study/NCT05570695) and the Stage 1 protocol was published (https://osf.io/qbkj2). The study was conducted in accordance with the latest version of the Declaration of Helsinki and recent consensus guidelines for neurofeedback studies [17] (see osf.io for the corresponding CRED-nf checklist). Prior to any experimental procedures, all participants provided written informed consent. The study entailed 2 sessions and participants received a fixed financial compensation of 25 € plus a variable, performance-based compensation of up to 25 € per session. Thus, participants received between 50 and 100 € in total.

### Sample composition and sampling plan

Using convenience sampling, a total of 124 participants were screened and 97 healthy, right-handed participants aged between 18 and 45 years with a good command of German were recruited for the study (see Fig H in S1 Text for a Consolidated Standards of Reporting Trials (CONSORT) flow diagram). Exclusion criteria comprised pregnancy, neurological or psychiatric diseases, severe internal diseases including diabetes, skin diseases, current or recurrent pain, regular intake of medication (aside from contraception, thyroidal, and antiallergic medication), previous surgeries at the head or spine, any previous side effects associated with thermal stimulation, contact to a person with a SARS-CoV-2 infection within the last 2 weeks, and current symptoms of a cold or flu. To be included in the analyses, participants had to be right-handed according to the short version of the Edinburgh handedness inventory (cut-off score: laterality quotient >60) [36], attend both sessions, and comply with instructions throughout the experiment. In addition, pain ratings were screened for floor and ceiling effects during the first session. Participants with average pain ratings below 5 or above 95 prior to neurofeedback (pain_pre_ assessment, see below) were excluded from the analyses and their participation was terminated prematurely (*n* = 5). Eighty-five participants completed session 1, of which 78 also completed session 2. After exclusion of 3 more participants (for reasons, see Fig H in S1 Text), the final sample comprised 75 participants (32 male, mean age 26 ± 6 years).

The sample size was determined by a sequential Bayesian sampling plan that defines a maximal sample size [37]. Specifically, we planned to continuously analyze incoming data using Bayesian statistics until compelling evidence for an effect or its absence (BF_01_ or BF_10_ ≥ 10) was found for specified primary analyses (see Table 1) or until a maximum of 95 participants had been included in our primary analyses [33,37]. The maximal sample size N_max_ = 95 was defined due to resource limitations and was determined using a Bayes factor design analysis (BFDA) for Bayesian paired samples *t* tests [37]. As evidence for an effect was found for the specified primary analyses at a sample size of *n* = 75, the stop criterion was reached and we concluded data collection at this point.

**Table 1:** Design table.

Hypotheses	Sampling Plan	Analysis Plan	Interpretation
**Q1: Are alpha oscillations up- and down-regulated?**			
**H1: Alpha oscillations are up- and down-regulated.** **H1.1: Time up/down-regulates alpha oscillations.** H1.1.a: AAI_ART_verum_2ndhalf_ > AAI_ART_verum_1sthalf_ H1.1.b: AAI_ALT_verum_2ndhalf_ < AAI_ALT_verum_1sthalf_ **H1.2: Neurofeedback up/down-regulates alpha oscillations.** If time effect: use averages across second half of trials (data_2ndhalf) Otherwise: use averages across all trials (data_total) H1.2.a: AAI_ART_verum_ > AAI_ART_sham_ H1.2.b: AAI_ALT_verum_ < AAI_ALT_sham_ **H1.3: Attention up/down-regulates alpha oscillations.** If neurofeedback effect: H1.3.a: AAI_ART_sham_ > AAI_ALT_sham_ Otherwise: H1.3.a: AAI_ART_avg(verum,sham)_ > AAI_ARLT_avg(verum,sham)_	SBF+maxN design with the following parameters: Starting at N_min_ = 20, the sample size will be increased with n_step_size_ = 5 until: • BF_10_ ≥ 10 or ≤ 1/10 for H1.2 (a or b) AND H1.3 or • N_max_ = 95 is reached. Note that the precise comparison for the evaluation of H1.3.a depends on the previous evaluation of H1.1 (time effects) and H1.2 (neurofeedback effects).	One-sided Bayesian paired samples *t* tests (Cauchy prior distribution with scale parameter r = √(2)/2 and truncation at zero) or one-sided Bayesian Wilcoxon signed rank tests (Cauchy prior distribution with scale parameter r = √(2)/2 and truncation at zero, 1,000 iterations) if normality assumptions are violated according to Q-Q-plot.	**H1: Alpha oscillations are up- and down-regulated.** If time AND neurofeedback AND attention effect: evidence for up- and down-regulation according to pattern 1 If time AND attention effect: evidence for up- and down-regulation according to pattern 2 If neurofeedback AND attention effect: evidence for up- and down-regulation according to pattern 3 If attention effect: evidence for up- and down-regulation according to pattern 4 If evidence for absence of attention effect: evidence for absence of up- and down-regulation Otherwise: inconclusive evidence **H1.1: Time up/down-regulates alpha oscillations.** If BF_10_ ≥ 3 for H1.1.a OR H1.1.b: evidence for time effect If BF_10_ ≤ 1/3 for H1.1.a AND H1.1.b: evidence for absence of time effect Otherwise: inconclusive evidence **H1.2: Neurofeedback up/down-regulates alpha oscillations.** If BF_10_ ≥ 3 for H1.2.a OR H1.2.b: evidence for neurofeedback effect If BF_10_ ≤ 1/3 for H1.2.a AND H1.2.b: evidence for absence of neurofeedback effect Otherwise: inconclusive evidence **H1.3: Attention up/down-regulates alpha oscillations.** If BF_10_ ≥ 3 for H1.3.a: evidence for attention effect If BF_10_ ≤ 1/3 for H1.3.a: evidence for absence of attention effect Otherwise: inconclusive evidence
**Q2: Are the perception of pain and underlying brain responses up- and down-regulated?**			
**H2: The perception of pain and underlying brain responses are up- and down-regulated.** **H2.1: Neurofeedback up/down-regulates pain ratings.** If AAI time effect: use averages across second half of trials (data_2ndhalf) to evaluate neurofeedback effect Otherwise: use averages across all trials (data_total) H2.1.a: rating_ART_verum_ < rating_ART_sham_ H2.1.b: rating_ALT_verum_ > rating_ALT_sham_ **H2.2: Attention up/down-regulates pain ratings.** If AAI neurofeedback effect: H2.2.a: rating_ART_sham)_ < rating_ALT_sham)_ Otherwise: H2.2.a: rating_ART_avg(verum,sham)_ < rating_ART_avg(verum,sham)_ **H2.3: Neurofeedback up/down-regulates brain responses.** Evaluated for each brain response (BR) separately. If AAI time effect: use averages across second half of trials (data_2ndhalf) to evaluate neurofeedback effect Otherwise: use averages across all trials (data_total) H2.3.a: BR_ART_verum_ < BR_ART_sham_ H2.3.b: BR_ALT_verum_ > BR_ALT_sham_ **H2.4: Attention up/down-regulates brain responses.** Evaluated for each brain response (BR) separately. If AAI neurofeedback effect: H2.4.a: BR_ART_diff(verum,sham)_ < BR_ALT_diff(verum,sham)_ Otherwise: H2.4.a: BR_ART_avg(verum,sham)_ < BR_ART_avg(verum,sham)_	The sample size for this analysis will be determined by the analysis of H1.3.a.	One-sided Bayesian paired samples *t* tests (Cauchy prior distribution with scale parameter r = √(2)/2 and truncation at zero) or one-sided Bayesian Wilcoxon signed rank tests (Cauchy prior distribution with scale parameter r = √(2)/2 and truncation at zero, 1,000 iterations) if normality assumptions are violated according to Q-Q-plot.	**H2: The perception of pain and underlying brain responses are up-and down-regulated.** Evaluated for pain ratings and each brain response separately. If time AND neurofeedback AND attention effect: evidence for up- and down-regulation according to pattern 1 If time AND attention effect: evidence for up- and down-regulation according to pattern 2 If neurofeedback AND attention effect: evidence for up- and down-regulation according to pattern 3 If attention effect: evidence for up- and down-regulation according to pattern 4 If evidence for absence of attention effect: evidence for absence of up- and down-regulation Otherwise: inconclusive evidence **H2.1: Neurofeedback up/down-regulates pain ratings.** If BF_10_ ≥ 3 for H2.1.a OR H2.1.b: evidence for neurofeedback effect If BF_10_ ≤ 1/3 for H2.1.a AND H2.1.b: evidence for absence of neurofeedback effect Otherwise: inconclusive evidence **H2.2: Attention up/down-regulates pain ratings.** If BF_10_ ≥ 3 for H2.2.a: evidence for attention effect If BF_10_ ≤ 1/3 for H2.2a: evidence for absence of attention effect Otherwise: inconclusive results **H2.3: Neurofeedback up/down-regulates brain responses.** Evaluated for each brain response separately. If BF_10_ ≥ 3 for H2.3.a OR H2.3.b: evidence for neurofeedback effect If BF_10_ ≤ 1/3 for H2.3.a AND H2.3.b: evidence for absence of neurofeedback effect Otherwise: inconclusive evidence **H2.4: Attention up/down-regulates brain responses.** Evaluated for each brain response (BR) separately. If BF_10_ ≥ 3 for H2.4.a: evidence for attention effect If BF_10_ ≤ 1/3 for H2.4.a: evidence for absence of attention effect Otherwise: inconclusive evidence
**Q3: Do alpha oscillations mediate attention effects on pain perception?**			
**H3.1:** A**lpha oscillations partially mediate attention effects on pain perception (mediation effect).** H3.1.a: 0 ∉ 95% CI ab_verum_ AND 0 ∉ 95% CI c’_verum_ (partial mediation effect in verum conditions) H3.1.b: 0 ∉ 95% CI ab_sham_ AND 0 ∉ 95% CI c’_sham_ (partial mediation effect in sham conditions) **H3.2: Mediation effects are stronger for verum compared to sham neurofeedback (moderation effect).** H.3.2.a: ab_verum_ > ab_sham_	The sample size for this analysis will be determined by the analysis of H1.3.a.	Mediation effect: Bayesian multilevel mediation analyses conducted separately for verum und sham conditions (X: ART_NF_ and ALT_NF_ (verum model) or ART_sham_ and ALT_sham_ (sham model); M: single-trial AAIs; Y: single-trial pain ratings). Moderation effect: One-sided Bayesian paired samples *t* tests (Cauchy prior distribution with scale parameter r = √(2)/2 and truncation at zero) or one-sided Bayesian Wilcoxon signed rank tests (Cauchy prior distribution with scale parameter r = √(2)/2 and truncation at zero, 1,000 iterations) if normality assumptions are violated according to Q-Q-plot.	**H3.1:** A**lpha oscillations partially mediate attention effects on pain perception (mediation effect).** If 0 ∉ 95% CI ab_verum/sham_ AND 0 ∉ 95% CI c’_verum/sham_ for H3.1.a/H3.1.b: evidence for partial mediation effect in verum/sham conditions If 0 ∉ 95% CI ab_verum/sham_ AND 0 ∈ 95% CI c’_verum/sham_ for H3.1.a/H3.1.b: evidence for full mediation effect in verum/sham conditions If 0 ∈ 95% CI ab_verum/sham_ for H3.1.a/H3.1.b: no evidence for mediation effect Note: Interpretability of mediation effect in verum condition is limited if no mediation effect is found in the sham conditions, because the causal sequence between X and M cannot be validated. **H3.2: Mediation effects are stronger for verum compared to sham neurofeedback (moderation effect).** If BF_10_ ≥ 3 for H3.2.a: evidence for stronger mediation effects for verum compared to sham neurofeedback If BF_10_ ≤ 1/3 for H3.2.a: evidence for equally strong mediation effects for verum compared to sham neurofeedback Otherwise: inconclusive evidence

AAI, alpha asymmetry index; ALT, attention left training; ART, attention right training; BF, Bayes factor; BR, brain response; SBF+maxN, sequential Bayes factor design with maximal N.

Bayesian paired samples *t* tests represent the main statistical test in our analysis pipeline (see Table 1, hypotheses 1 and 2) and are also used to establish moderation effects (see Table 1, hypothesis 3) in the moderated mediation analysis, for which no BFDA implementation exists at the time of writing. Thus, BFDAs for Bayesian paired samples *t* tests were used to determine a maximal sample size for all hypotheses. To account for potential non-responders [18,38], the BFDA was conducted with a weighted effect size estimate of Cohen’s d = 0.41 based on the following assumptions: 70% responders with an effect size estimate of Cohen’s d = 0.5 and 30% non-responders with an effect size estimate of Cohen’s d = 0.2. For responders, a medium effect size was chosen because this represents a standard effect size estimate used in translational neuroscience to detect effects which are behaviorally, and potentially clinically relevant. Non-responders were defined as participants with performance below a certain threshold (<50% successful trials [39]). Thus, the corresponding effect size estimate was set to Cohen’s d = 0.2 and not 0. To further validate our weighted effect size estimate, we summarized effect sizes reported in related short-term neurofeedback studies targeting alpha oscillations [30,31,40] (Table A in S1 Text). This analysis resulted in an average effect size estimate of Cohen’s d = 1.3 for neuronal effects indicating that our estimate is rather conservative. Since these estimates are subject to uncertainty, BFDAs for different effect size estimates were performed as well and can be found in the section “Bayes factor design analysis” below, along with further information on the analysis.

#### Bayes factor design analysis

To determine the maximal sample size for a sequential Bayes factor design with maximal N (SBF+Nmax) and to evaluate the properties of such a design, we conducted a Bayes factor design analysis [37,41]. To this end, we first computed an open-ended SBF design for one-sided paired *t* tests under both H1 and H0, yielding a distribution of sample sizes at the stopping point for both hypotheses (parameters: Cohen’s d H1 = 0.41, Cohen’s d H0 = 0, Cauchy prior distribution with scale parameter r = √(2)/2 and truncation at zero, directionality H1 = “greater”, minimal sample size = 20, step size = 5, maximal sample size = 300, lower BF10 boundary = 1/10, upper BF10 boundary = 10, number of simulated studies = 10,000. Note that the maximal sample size for simulations was set to 300 to explore the development of power across a wide range of sample sizes). Subsequently, we determined the sample size at which 90% of studies reached the defined BF10 boundaries (1/10 or 10) under the H1. The resulting sample size was then used to evaluate a corresponding SBF+N_max_ design with respect to false positives and false negatives rates.

Fig A in S1 Text visualizes the results of an SBF+N_max_ design with the obtained N_max_ = 95. Under H0, 86.0% of simulated studies correctly indicated evidence for the absence of an effect (58.3% at BF_10_ boundary = 1/10 + 27.7% at the BF_10_ boundary = 1/3), while the false positive rate was acceptably low (2.3%). The average sample size at the stopping point was 68. Under H1, 95.6% of simulated studies correctly indicated evidence for the presence of an effect (90.9% at BF_10_ boundary = 10 + 4.7% at the BF_10_ boundary = 3), while the false negative rate was acceptably low (0.3%). The average sample size at the stopping point was 49. Thus, the chances of detecting an effect, if it exists, were rated as sufficiently high for the proposed SBF+N_max_ = 95 design.

Since effect size estimates (ES) are subject to uncertainty, we explored the effect of our assumptions by conducting BFDAs for further ES. The performance of a sequential Bayes Factor design with different ES and sample sizes is illustrated in Fig B in S1 Text.

### Design

#### Experimental procedure

**Fig 2:**
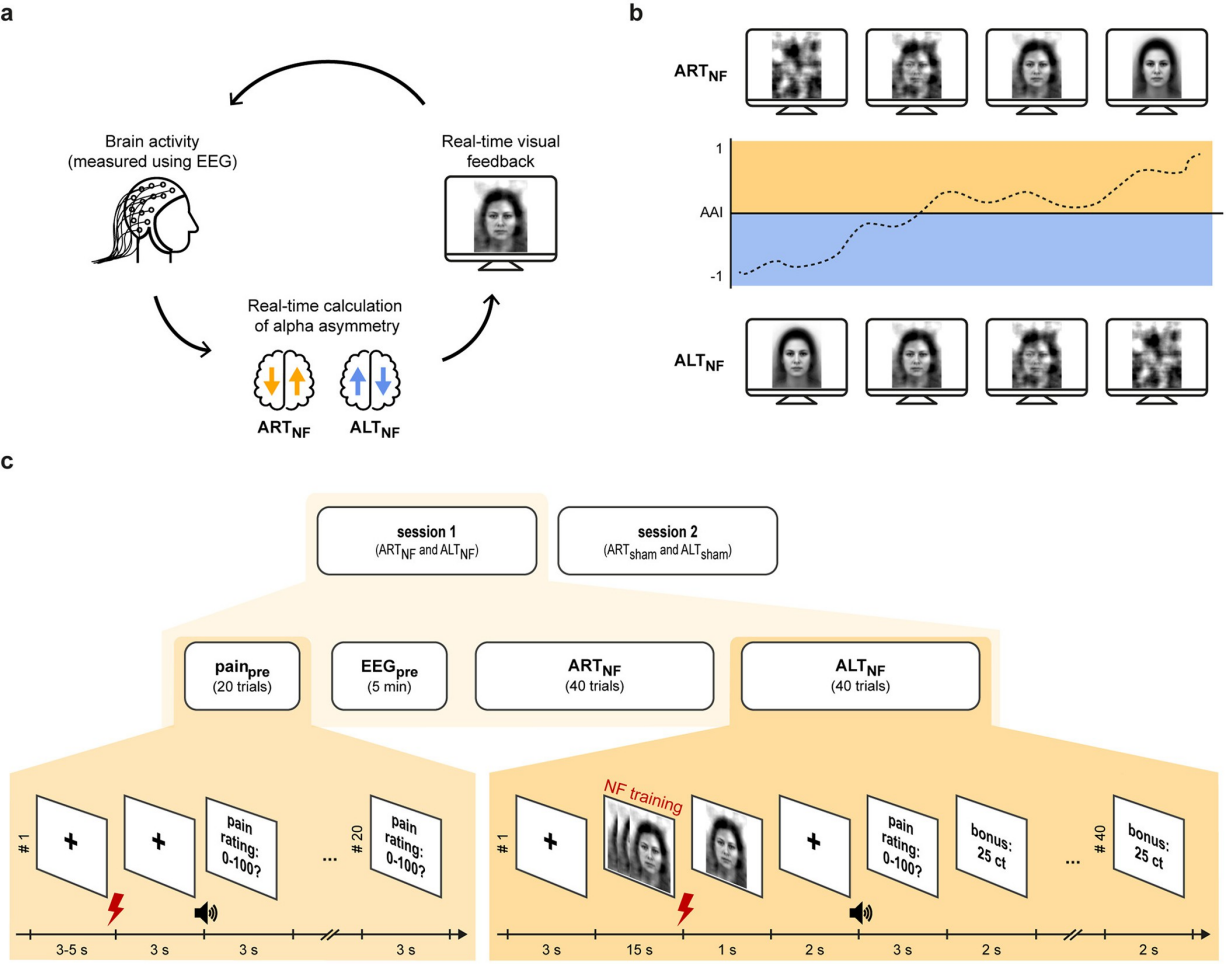
Experimental procedure. (a) Attention-based neurofeedback training. During neurofeedback, the somatosensory AAI was calculated in real-time based on 1,000 ms EEG data segments and was fed back to participants using the visibility of neutral face images [44]. This feedback cycle was updated every 100 ms. To control for nonspecific effects, the experiment entailed 2 verum neurofeedback conditions during which alpha asymmetry was regulated in opposite directions as well as 2 sham neurofeedback conditions with identical instructions but yoked feedback. During the attention right training (ART_NF_), participants were instructed to focus attention on their right hand and the up-regulation of right relative to left alpha oscillations was incentivized. During the attention left training (ALT_NF_), participants were instructed to focus attention on their left hand and the down-regulation of right relative to left alpha oscillations was incentivized. (b) Real-time visual feedback. AAI values and corresponding feedback signals for the verum conditions are displayed. For ART, AAI values above −0.6 led to an increase of image visibility until full visibility was reached at an AAI of 0.6. For ALT the relationship between AAI and image visibility was reversed. In addition, a small, central fixation cross was superimposed on the images to support image fixation. (c) Experimental design. Verum and sham neurofeedback conditions were completed in a pseudo-randomized order during 2 separate sessions. Each session entailed verum or sham conditions (ALT_NF_ and ART_NF_ or ALT_sham_ and ART_sham_, 40 trials each) as well as baseline assessments of pain sensitivity (pain_pre_, 20 trials) and brain activity (EEG_pre_, 5 min resting state with eyes open). The latter, baseline measures of pain sensitivity and brain activity, were obtained to evaluate predictors of regulatory success and will be analyzed and reported in a separate manuscript. The sequence of events of neurofeedback trials is shown on the right. After a fixation period of 3 s, the regulation period of 15 s began. Immediately afterwards, a noxious stimulus was applied. To avoid that pain-related brain responses were confounded by visual offset responses, the last feedback signal of the regulation period remained on the screen for another second before turning into a fixation cross. Two seconds later, an auditory and visual cue prompted participants to rate the perceived pain intensity. Finally, the financial reward earned on a given trial was displayed. The face stimulus shown as an example for the visual feedback is derived from [44], image ID: FNES. AAI, alpha asymmetry index; ALT, attention left training; ART, attention right training; EEG, electroencephalography; NF, neurofeedback.

Methodology and terminology of this study are based on recent recommendations for EEG and neurofeedback studies [17,42,43]. Our EEG-based neurofeedback study employs a within-subjects, bidirectional control design with 2 verum and 2 sham conditions which were completed during 2 sessions (Fig 2). The design was adapted from recent neurofeedback studies which successfully modulated the asymmetry of alpha oscillations [30,31]. In a first verum neurofeedback condition, participants were instructed to focus attention on their right hand and the up-regulation of alpha oscillations in the right hemisphere relative to alpha oscillations in the left hemisphere was incentivized through neurofeedback (attention right training, ART_NF_). In a second verum neurofeedback condition, participants were instructed to focus attention on their left hand and the down-regulation of right relative to left alpha oscillations was incentivized (attention left training, ALT_NF_). To control for nonspecific effects of the neurofeedback procedure such as expectation effects evoked by the explicit instruction, the 2 verum conditions were accompanied by 2 matched sham neurofeedback conditions administered in a separate session. During the sham neurofeedback conditions, participants obtained the same instructions, i.e., focus attention on your right hand (sham attention right training, ART_sham_) or focus attention on your left hand (sham attention left training, ALT_sham_). However, the feedback signal did not mirror their brain activity. Instead, the feedback signal and the corresponding reward of the last matching verum condition completed by a previous participant, i.e., ART_NF_ for ART_sham_ and ALT_NF_ for ALT_sham_, were replayed (yoked feedback). Thus, on a group level, feedback signal and compensation were identical between verum and sham conditions supporting blinding of participants and experimenters. To implement sham conditions for the first participant(s), data from a “participant zero” was collected, who only completed the verum trainings and was excluded from the analyses. To avoid order effects, sessions were separated by 7 days at least and the order of conditions between (verum versus sham) and within sessions (ART versus ALT) was pseudo-randomized.

Both sessions comprised a fixed sequence of events aimed at capturing changes of alpha asymmetry, pain perception, and pain processing that occur during the neurofeedback training (Fig 2). Each experimental condition entailed 40 trials with 15 s of neurofeedback training, which were followed by a brief noxious laser stimulus applied to the dorsum of the left hand. Three seconds after the noxious stimulus, participants verbally rated the perceived pain intensity on a numerical rating scale (NRS) ranging from 0 (“no pain”) to 100 (“worst tolerable pain”). To enhance motivation, participants received feedback regarding their performance-based financial compensation at the end of each trial. Thus, the total neurofeedback training time per condition was 10 min. Alpha asymmetry during these training runs served as read-out of regulation success. Pain ratings served as read-out for pain perception. Brain responses to noxious stimuli served as read-out for pain processing. To quantify baseline measures, pain perception, pain processing, and resting-state brain activity were assessed before (pain_pre_ and EEG_pre_, respectively) the neurofeedback training. During the pain_pre_ assessment, participants rated 20 noxious laser stimuli applied to the left hand on the same NRS used during neurofeedback and pain ratings served as read-out for pain perception. Brain responses to noxious stimuli served as read-out for pain processing. The EEG_pre_ assessment entailed 5 min of resting-state recordings with eyes open during which a central fixation cross was displayed and served as read-out for the brain state during rest.

Throughout the experiment, participants were seated in a comfortable chair facing a computer screen. To minimize distraction, participants were exposed to white noise via headphones and opaque cloth prevented them from seeing their left hand or the experimenter handling the laser device. To familiarize participants with the noxious laser stimulation, 5 training trials including noxious stimulation and subsequent pain ratings were performed before the pain_pre_ assessment at both sessions. In addition, a 30 s neurofeedback familiarization was performed before the first neurofeedback condition in the first session. During this time, participants were instructed to passively watch the real-time feedback signal, which mirrored their alpha asymmetry.

#### Blinding

Double-blinding was enabled through the usage of sham conditions and participant-specific numeric codes encoding the order of conditions. During each session, these codes automatically (1) determined the predefined training conditions; and (2) generated file names. Thus, the experimenter was blinded during data acquisition as well as during subsequent preprocessing and analysis steps. To ensure blinding of the participants, we additionally provided a cover story. Specifically, we informed participants that the experiment would investigate how 2 different neurofeedback trainings affected pain perception and whether training effects fluctuated over time. We did not provide any information regarding the underlying feedback feature or our study hypotheses until the debriefing at the end of the last session. Since both verum and sham conditions comprised an identical sequence of events and resemble each other with respect to the feedback signal and reward, expectation effects which match our experimental hypotheses are highly unlikely. To evaluate blinding, participants were asked to rate how well they could regulate the visibility of faces every 20 trials, i.e., twice per condition, on a visual analogue scale (VAS) ranging from 0 (“not at all”) to 100 (“very well”). In addition, participants were asked to indicate whether they completed verum or sham neurofeedback during session 2 after debriefing. Prespecified control analyses (see below) assessing the impact of unblinding on the results of primary analyses confirmed that the blinding success did not vary systematically between ART and ALT conditions (BF_10_ = 0.13 and 0.13, respectively).

#### EEG recording

Brain activity was recorded using BrainAmp MR plus amplifiers and 64 actiCAP snap sensors placed according to the 64-channel extended international 10–20 system (Brain Products GmbH, Gilching, Germany). All sensors were referenced to FCz and grounded at FPz. In addition, electrooculographic activity was recorded with a bipolar Brain Amp ExG MR amplifier (Brain Products GmbH, Gilching, Germany) and Ag/AgCl sensors placed below the outer canthus of each eye. An Ag/AgCl sensor attached at the nasion served as ground. All recordings were performed at a sampling rate of 500 Hz (0.2 μV resolution) and band-pass filtered between 0.016 and 250 Hz. Impedances were measured directly before the pain_pre_ run and sensors were prepared until impedances below 20 kΩ were achieved for all active and passive sensors. Pain ratings were manually added to the EEG data as markers during the experiment.

#### Noxious stimulation

Noxious stimuli were applied using cutaneous laser stimulation, which enables the well-controlled stimulation of nociceptive pathways without concomitant stimulation of tactile pathways [45]. All stimuli were applied to the dorsum of the left hand using a neodymium:yttrium-aluminum-perovskite (Nd:YAP) laser (Stimul 1340, DEKA M.E.L.A. srl, Calenzano, Italy) and the following settings. Stimulus duration was set to 4 ms and stimulus diameter to 7 mm. Laser intensity was set to 3.5 J, which induces stable brain responses while being well tolerated [46]. To avoid tissue damage and minimize habituation/sensitization effects, stimulation sites were changed slightly after each stimulus. For safety reasons, both study personal and participants wore safety goggles throughout the experiment.

#### Verum neurofeedback training

Motivated by recent studies [30,31,40], we designed a short-term neurofeedback protocol with real-time data analysis and feedback visualization using MATLAB (version: R2020b, Mathworks, Natick, Massachusetts, United States of America) and the MATLAB-based toolboxes FieldTrip (version: 20210212, [47]) and Psychtoolbox-3 (version: 3.0.17 beta, https://psychtoolbox.org). To this end, EEG data was streamed from an acquisition computer (operating system: Windows 10) to a second computer (operating system: Ubuntu 20.04.2 LTS) responsible for data processing and stimulus presentation in real-time and is stored in a buffer. Every 100 ms, 1,000 ms segments were accessed from this buffer and analyzed as follows. Data were demeaned, and power estimates from 8 to <13 Hz were obtained with a 1 Hz resolution using a Hanning-tapered Fast Fourier Transformation. Subsequently, alpha power over right and left somatosensory regions was calculated by averaging across 8 to 12 Hz and the channels C4, CP4, CP6 and C3, CP3, CP5, which overlie the somatosensory cortex and yield a higher signal-to-noise ratio than single channels [48]. Resulting power estimates were then used to calculate the alpha asymmetry index (AAI), defined as:

$$\mathrm{AAI}(t)=\frac{\alpha_{rS1}(t)-\alpha_{lS1}(t)}{\alpha_{rS1}(t)+\alpha_{lS1}(t)},$$

where α_rS1_(*t*) and α_lS1_(*t*) represent alpha power in time window *t* in right and left somatosensory regions, respectively. AAI values range from −1 to 1 when power is purely left- or right-lateralized, respectively, and were communicated to participants by altering the visibility of neutral face images (see paragraph below and section “Image visibility” in the Supporting information for details).

Participants were instructed to use spatial attention towards the left or right hand to enhance the visibility of face images as much and as long as possible until the pain stimulus is applied [30]. To enhance motivation, participants received a performance-based financial compensation for each trial. This compensation reflected the average AAI on a given trial and amounted to up to 0.25 € per trial (ART: linear increase from 0–0.25 € for positive AAI values with a bonus of 0.25 if the AAI was larger than 0.6; ALT: linear increase from 0–0.25 € for negative AAI values with a bonus of 0.25 if the AAI was smaller than −0.6).

#### Visual feedback signal

To create a motivating feedback visualization while avoiding potential effects of emotion or gender, AAI values were fed back to participants by altering the visibility of neutral face images presented centrally on a screen. Specifically, 8 neutral face images depicting an average female and male face from 4 different angles were taken from the Averaged Karolinska Directed Emotional Faces (AKDEF) data set [44] (image IDs: F/MNEFL, F/MNEFR, F/MNEFHL, F/MNEHR; see Fig C in S1 Text for a visualization). For each trial, one of these images was randomly chosen, thus avoiding systematic effects linked to one of the images. Depending on the training condition, image visibility was altered in opposite directions. During ART_NF_, AAI values above −0.6 led to an increase of image visibility until full visibility was reached at an AAI of 0.6. During ALT_NF_, AAI values below 0.6 led to an increase of image visibility until full visibility was reached at an AAI of −0.6 (see below section “Image visibility” and Fig C in S1 Text for details). During sham conditions, the feedback signal of the last verum condition completed by another participant was replayed (yoked feedback) irrespective of the current alpha asymmetry.

#### Image visibility

Let *c* be the parameter controlling visibility. It ranges from zero (no visibility) to one (full visibility) and can be defined as a function of the AAI and the condition ALT/ART as follows:

$$c=\{\begin{array}{ll} 1 & \mathrm{if}(\mathrm{ART}\wedge 0.6<AAI)\vee (\mathrm{ALT}\wedge AAI<-0.6)\\ 0.2+\frac{4}{3}|AAI| & \mathrm{if}(\mathrm{ART}\wedge 0<AAI<0.6)\vee (\mathrm{ALT}\wedge -0.6<AAI<0)\\ 0.2-\frac{1}{3}|AAI| & \mathrm{if}(\mathrm{ART}\wedge -0.6<AAI<0)\vee (\mathrm{ALT}\wedge 0<AAI<0.6)\\ 0 & \mathrm{if}(\mathrm{ART}\wedge AAI<-0.6)\vee (\mathrm{ALT}\wedge AAI>0.6). \end{array}$$

To achieve different levels of visibility, a scrambled version of each image was created by randomly perturbing the phase angle of the 2D Fourier transformation of the original image. The magnitude of the perturbation is proportional to the visibility parameter *c* introduced above. In this manner, low-level image properties such as spatial power spectrum and luminance are held constant across varying AAI-values within a trial. The procedure of generating the scrambled images is explained in the following. Let $I\in R^{n_{x}\times n_{y}}$ be the matrix representation of a gray scale image with *n*_*x*_ by *n*_*y*_ pixels. Let 〈*I*〉 denote the mean value of the image. The 2D FFT of the demeaned image is given by

$$\hat{I}={\mathrm{FFT}}_{2D}\{I-\langle I\rangle \}.$$

The scrambled image is obtained as the inverse 2D FFT of the Hadamard product of $\hat{I}$ and a complex-valued perturbation matrix *P*(*c*):

$$\begin{array}{r} I_{\mathrm{scramb}}(c)={\mathrm{FFT}}_{2D}^{-1}\{\hat{I}⊙P(c)\}+\langle I\rangle \end{array}.$$

The entry in the k-th row and l-th column of ***P***(*c*) is

$${\left[P(c)\right]}_{kl}=\exp\left(i\;\Delta \phi_{kl}\;(1-c)\right),$$

where Δϕ_kl_ are random phase angles drawn from a uniform distribution on the interval [−π, π]. To ensure that the inverse 2D FFT is real-valued, the random phase angles satisfy the symmetry property ${\Delta \phi }_{kl}=-\Delta \phi_{n_{x}{-kn}_{y}-l}$. In the scrambling of the AKDEF images used in this study, the lowest frequency components of the image were excluded from the random phase perturbation. This was necessary since the scrambled images would otherwise show a pattern consisting of horizontal and vertical stripes.

To avoid sudden jumps in the feedback signal arising from rather fast fluctuations of the AAI (around 1 Hz), an online filter inducing temporal smoothing was applied to the AAI signal. The temporally smoothed AAI signal is defined as follows:

$$\begin{array}{r} \mathrm{fAAI}(t)=\int_{-\Delta \tau }^{t}\mathrm{AAI}(t-\tau )W(\tau )d\tau \end{array},$$

where *W*(τ) is a linear weighting function, i.e.,

$$W(\tau )=\left\{ \begin{array}{ll} \frac{2(\Delta \tau -\tau )}{\Delta \tau^{2}} & \mathrm{if}\;0\leq \tau \leq \Delta \tau \\ 0 & \mathrm{otherwise} \end{array}\right.$$

where Δτ is the temporal support of the weighting function. Note that since $\int_{-\infty }^{\infty }W(\tau )d\tau =1$, the fAAI signal represents a weighted average of the unfiltered AAI signal. As stated previously, the AAI signal is evaluated every 100 ms. Thus, in practice, only finitely many, discrete values of the AAI signal are available. Consequently, in the equation above, the AAI(.) function is substituted with a piecewise linear approximation that interpolates the discrete values.

#### Psychological measures and regulatory strategies

Following recent guidelines, psychological variables that might influence training performance and could thus predict regulatory success were assessed using self-report measures [17,49]. These analyses will be reported separately. At the beginning of each session, the motivation to participate in the training was assessed using a seven-point Likert scale ranging from 1 (“very low”) to 7 (“very high”). In addition, we assessed the general self-efficacy, the health-related locus of control, and the current positive or negative affect of participants at the beginning of each session. This was done using the German versions of the General Self-Efficacy Scale (GSE) [50], the Multidimensional Health Locus of Control (MHLC) Scales [51], and the Positive and Negative Affect Schedule (PANAS) [52]. After each condition, i.e., twice per session, the perceived task demand and the effort exerted was assessed using the same seven-point Likert scale as for motivation and potential regulatory strategies were investigated by asking participants to “describe in a few words which strategy/strategies you have used to regulate the feedback signal.”

### Analysis plan

#### Preprocessing

Before submitting data sets to the preprocessing pipeline, data were transformed to BIDS format [42] using the MATLAB-based (version: R2020b, Mathworks, Natick, Massachusetts, USA) toolbox FieldTrip (version: 20210212, [47]). Subsequent preprocessing of EEG data was conducted using the automatic preprocessing part of the DISCOVER-EEG pipeline [53–55]. In the Stage 1 manuscript of this Registered Report, we proposed a manual preprocessing pipeline. In consultation with the editorial board, and, importantly, before preprocessing any data sets, we switched from manual to automatic preprocessing, as this is the timelier method and enhances the objectivity, replicability, and speed of preprocessing. For each session, the preprocessing pipeline was applied to the concatenated neurofeedback data sets (ART_NF_ and ALT_NF_ or ART_sham_ and ALT_sham_). Thus, 2 data sets were preprocessed for every participant. For every data set, intervals from −18 to 2 s with respect to laser stimuli were selected and concatenated for preprocessing. Preprocessing included line noise removal, bad channel detection and interpolation, re-referencing to the average reference, independent component analysis (ICA), and the automatic detection of bad segments. Prespecified control analyses (see below) assessing the impact of artefacts on the results of primary analyses confirmed that the amount of artefacts did not vary systematically between conditions (BF_10_ = 0.41 and 0.13, respectively). Further analyses were performed using FieldTrip along with custom written code.

#### Primary analyses

Primary analyses assessed the effects of our neurofeedback training in sensor space. Specifically, we examined distinct result patterns capturing attention, neurofeedback, and time effects (Fig 1) on alpha oscillations (hypothesis 1) as well as pain perception and underlying brain responses (hypothesis 2). In addition, we aimed to employ moderated multilevel mediation analysis to test whether changes in attention, alpha oscillations, and pain perception can be integrated into a single, mechanistic model (hypothesis 3).

*Hypothesis 1: Alpha oscillations are up- and down-regulated.* To examine changes in alpha oscillations, AAIs were extracted from the preprocessed neurofeedback data sets following the same procedure as during neurofeedback. For each trial, data from the last 12 s of the training period were extracted to limit the impact of the smoothing function used for AAI visualization (see section “Image visibility” in the Supporting information for details). Then, data were segmented into 1,000 ms epochs with 900 ms overlap and epochs with artefacts that had been marked during preprocessing were rejected (see section “Preprocessing” for details on applied artefact criteria). Remaining epochs were demeaned and power estimates from 8 to <13 Hz were obtained with a 1 Hz resolution using a Hanning-tapered Fast Fourier Transformation. For every epoch, alpha power was quantified for right and left somatosensory regions by averaging across frequencies (8 to 12 Hz) and channels (C4, CP4, CP6 and C3, CP3, CP5, respectively) and alpha asymmetry indices were calculated. Finally, single trial alpha asymmetry indices were obtained by averaging asymmetry indices across all epochs per trial.

Subsequently, asymmetry indices were analyzed using an adaptive analysis pipeline which allowed us to differentiate between 4 response patterns describing attention, neurofeedback, and time effects (see Table 1 and Fig D in S1 Text for a summary and visualization of the pipeline). As a basis for these analyses, single trial asymmetry indices of the first and second half of each condition (≙ 20 trials) were averaged separately for every participant resulting in 8 averages per participant (2 time periods × 4 conditions). To examine changes in alpha asymmetry over time (time effect; H1.1), single-subject first and second half averages were compared separately for ART_NF_ and ALT_NF_ conditions. If at least one comparison yielded a time effect (increase for ART_NF_ and a decrease for ALT_NF_, respectively), second half averages were used as basis for all subsequent analyses. If no time effect was found, first and second half averages were averaged, and the resulting values were used for subsequent analyses. To examine whether verum neurofeedback led to larger increases/decreases in alpha asymmetry than sham neurofeedback (neurofeedback effect; H1.2), single-subject asymmetry averages from the verum conditions were compared to the corresponding sham conditions. If at least one comparison yielded a neurofeedback effect, data of the sham conditions would be used to evaluate whether ART and ALT conditions yielded opposite patterns of brain activity (attention effect; H1.3). Otherwise, data from corresponding verum and sham conditions was averaged, and the resulting single-subject ART and ALT average scores were compared.

*Hypothesis 2: The perception of pain and underlying brain responses are up- and down-regulated.* To examine changes in pain perception, pain ratings were extracted from the preprocessed neurofeedback data sets and were analyzed by repeating the analysis steps used for alpha asymmetry indices (including decisions on data selection, see Table 1 and Fig E in S1 Text for a summary and visualization). Since alpha oscillations and pain ratings are inversely related, the direction of pairwise comparisons was reversed, however. Subsequently, neurofeedback effects (H2.1) and attention effects (H2.2) on pain ratings were evaluated. Time effects were not analyzed due to confounding effects of habituation or sensitization.

Correspondingly, neurofeedback (H2.3) and attention effects (H2.4) were evaluated for brain responses which are typically observed after a noxious stimulus (see Table 1 and Fig F in S1 Text for a summary and visualization). These include a characteristic sequence of evoked potentials referred to as N1, N2, and P2 responses [56,57]. In addition, noxious stimuli suppress neuronal oscillations in the alpha (8 to <13 Hz) and beta (13 to 30 Hz) frequency bands and induce oscillations in the gamma (30 to100 Hz) frequency band [1,2]. Single-trial evoked and oscillatory brain responses to noxious stimuli were quantified using established procedures [46,58] which have been validated on a published data set ([59]; Fig F in S1 Text). To examine evoked brain responses, preprocessed data from the neurofeedback runs was bandpass filtered between 1 and 30 Hz (fourth-order Butterworth). Then, a baseline correction was applied using the fixation period preceding the neurofeedback training. Specifically, amplitudes from 2,000 to 2,500 ms with respect to the beginning of the fixation period were subtracted from the post stimulus data. Subsequently, evoked responses were quantified in a two-step procedure. First, individual peak latencies of evoked responses were determined based on averages across all trials of all neurofeedback conditions. To this end, local minima/maxima of the averaged waveform were determined at predefined channels (N1: C4, N2: Cz; P2: Cz) [46,58] and in predefined time-windows (N1: 120–200 ms; N2: 180–300 ms; P2: 250–500 ms) [46]. Second, single-trial amplitudes were obtained by averaging across a 30 ms window [58] centered at the previously defined peak latency. To quantify the N1 response, data were additionally re-referenced to Fz [60] before calculating the average waveform across all trials. To examine oscillatory brain responses, preprocessed data from the neurofeedback runs were filtered (fourth-order Butterworth 1 Hz high-pass filter, 41 to 51 Hz band-stop filter to dampen line noise). Next, single trial time-frequency estimates were obtained using a Hanning-tapered Fast Fourier Transformation and a sliding window approach. To obtain alpha and beta responses, a sliding window with a length of 500 ms and a step size of 20 ms was used. To obtain gamma responses, the window length was shortened to 250 ms, while the step size remained 20 ms. Finally, responses at alpha and beta frequencies were assessed by calculating the mean power across 8 to 12 Hz and 14 to 30 Hz, respectively, across a time window of 500 to 900 ms [46] and across the channels Cz, CPz, C2, C4, CP2, and CP4 [46]. Responses at gamma frequencies were assessed by calculating the mean power across 70 to 90 Hz [58], a time window of 150 to 350 ms [58] at Cz [46]. Brain activity in the theta frequency band was not analyzed as it mainly represents time-locked activity, which is captured by the laser evoked potential analyses [1,61].

*Hypothesis 3: Changes in alpha oscillations mediate attention effects on pain perception.* To link changes of alpha oscillations to changes in pain perception on a single-trial level, we employed a moderated multilevel mediation analysis based on data from all neurofeedback conditions. Mediation models examine whether the covariance between 2 variables (X and Y) can be explained by an intermediate variable termed mediator (M). Additional moderators make it possible to assess whether the strength of mediation effects varies across different conditions or groups [62]. Applied to the current study, such analyses allow us to assess whether the relationship between ART and ALT neurofeedback conditions (X) and single-trial pain ratings (Y) can be explained by the AAI (M) on a given trial and whether this effect is more pronounced for verum than for sham neurofeedback (moderator) (H3). To this end, the bi-variate relationships between X, M, and Y variables were quantified, and the mediation effect was determined based on the resulting path coefficients. Finally, path coefficients and the mediation effect can be compared between different levels of the moderator. Together, this procedure yields 2 advantages. First, mediation analyses quantify the relationship between single-trial brain activity (AAIs) and behavior (pain ratings) which is increasingly analyzed by neurofeedback studies [17]. Second, mediation analyses go beyond bi-variate brain–behavior analyses by integrating all variables of interest (i.e., attention, AAIs, and pain ratings) into a single model and thereby fosters mechanistic insights.

*Data exclusion criteria for primary analyses.* To ensure data quality, the following exclusion criteria were applied for primary analyses. First, single trials were omitted from all analyses if (1) more than 50% of data segments from the neurofeedback training period were rejected due to artefacts; or (2) no/an invalid pain rating (outside to the range of 0 to 100) occurred. Second, participants (3) with less than 10 remaining trials for a neurofeedback condition (*n* = 1); or (4) focusing on the wrong hand were omitted from all primary analyses. In addition, trials were removed from the analysis of evoked and oscillatory responses, if artefacts were detected during time periods used for baseline correction or the 1,500 ms time interval following the laser stimulus.

#### Control analyses

Control analyses assessed the impact of artefacts and unblinding on the results of primary analyses [17] by examining whether artefacts and blinding varied systematically between ART and ALT conditions. To this end, we calculated single-subject artefact (AI) and blinding indices (BI) for ART and ALT conditions and compared the resulting values between conditions. The A/BIs were defined as follows:

$${AI}_{AR/LT}=\frac{{trials}_{AR/LT\_NF}-{trials}_{AR/LT\_sham}}{{trials}_{AR/LT\_NF}+{trials}_{AR/LT\_sham}}$$

and

$${BI}_{AR/LT}=\frac{{blinding}_{AR/LT\_NF}-{blinding}_{AR/AL\_sham}}{{blinding}_{AR/AL\_NF}+{blinding}_{AR/AL\_sham}}$$

with trials_AR/LT_NF/sham_ representing the number of trials rejected in the respective condition and blinding_AR/LT_NF/sham_ representing the average blinding score in the respective condition.

#### Statistics

All statistical analyses were conducted in the R programming environment (version: 4.1.1, [63]). Pair-wise comparisons were conducted using one-sided, Bayesian parametric paired samples *t* tests or nonparametric signed rank tests depending on the data distribution. For parametric *t* tests, the “BayesFactor” package was used (version: 0.9.12–4.2, [64], parameters: Cauchy prior distribution with a scale parameter r = √(2)/2 and truncation at zero). For nonparametric rank tests, the functions “signRankGibbsSampler” and “computeBayesFactorOneZero” were used ([65], parameters: Cauchy prior distribution with a scale parameter r = √(2)/2 and truncation at zero 1,000 iterations). Resulting Bayes factors (BF_10_) are reported along with corresponding effect size estimates quantifying the median of the posterior Cohen’s δ distribution and its 95% credibility interval. Thereby, tests quantify evidence for both the presence and absence of effects [33].

Multilevel mediation analysis was conducted using the “bmlm” package (version: 1.3.4, [66,67]). Using Bayesian multilevel modeling and Markov chain Monte Carlo procedures, 5 path coefficients and corresponding confidence intervals were calculated for each participant (first-level coefficients) as well as on a group level (second-level coefficients). These coefficients represent the relationship between X and M (path a), the relationship between X and Y (path c), the relationship between M and Y controlled for X (path b), the relationship between X and Y controlled for M (path c’), and the mediation effect (path ab) [68]. To quantify moderation effects, we conducted 2 multilevel mediation analyses separately for verum and sham neurofeedback conditions. Subsequently, we planned to compare the resulting first-level coefficients between models separately for each path using the parametric or nonparametric tests described above under the condition that a mediation effect is found. Preceding the mediation analyses, X was recoded (ART, −1 and ALT, 1) and M (single-trial AAIs) was centered within-person [66].

## Results

### Alpha oscillations were up- and down-regulated by time, attention, and neurofeedback

**Fig 3:**
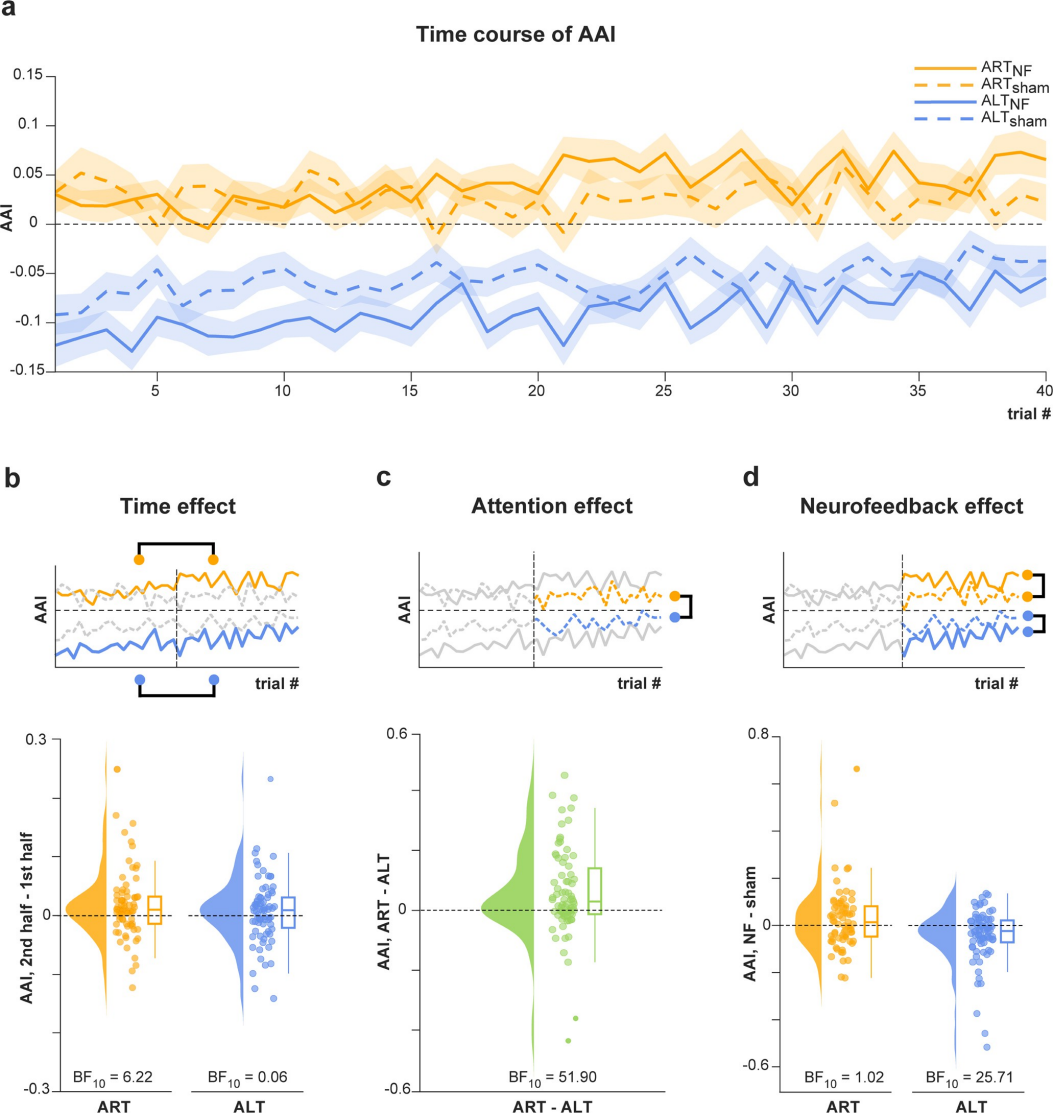
Observed result patterns characterizing time, attention, and neurofeedback effects on alpha oscillations. (a) Time course of the grand average AAI over trials, separately for the verum neurofeedback (solid lines) and sham conditions (dashed lines) and the ART (yellow lines) and ALT conditions (blue lines), respectively. (b) Effect of time on the AAI. The upper panel illustrates the analysis, which included the contrast of the mean AAI between the first and second half of trials, calculated separately for the ART_NF_ and ALT_NF_ conditions. The lower panel shows the difference (second half–first half of trials) of single-participant mean AAIs for the ART_NF_ (left) and ALT_NF_ (right) conditions. (c) Effect of attention on the AAI. The upper panel illustrates the analysis, which was performed on the second half of trials and included the contrast of the mean AAI between the ART_sham_ and ALT_sham_ conditions, respectively. The lower panel shows the difference of single-participant mean AAIs in the respective conditions. (d) Effect of neurofeedback on the AAI. The upper panel illustrates the analysis, which was performed on the second half of trials and included the contrast of the mean AAI between the verum and sham conditions, calculated separately for the ART and ALT conditions. The lower panel shows the difference (verum—sham) of single-participant mean AAIs for the ART (left) and ALT (right) conditions. Taken together, the observed data correspond most closely to predicted result pattern 1 (Fig 1), which incorporates an effect of attention, neurofeedback, and time on alpha asymmetry. Raincloud plots show un-mirrored violin plots displaying the probability density function of the data, individual data points, and boxplots. Boxplots depict the sample median, first (Q1), and third quartiles (Q3). Whiskers extend from Q1 to the smallest value within Q1–1.5* IQR and from Q3 to the largest values within Q3 + 1.5* IQR. The data underlying this figure can be found at https://osf.io/2mnys/. AAI, alpha asymmetry index; ALT, attention left training; ART, attention right training; BF, Bayes Factor; IQR, interquartile range; NF, neurofeedback.

We expected that lateralized attention to the left (ALT) or right (ART) hand would lead to an up-/down-regulation of alpha power appearing as a lateralization/asymmetry of alpha power across the left and right hemispheres (with alpha power being up-regulated ipsilateral to the attentional focus, down-regulated contralateral to the attentional focus, or a combination of both). Moreover, we expected these attention effects to be amplified by neurofeedback. In addition, before analyzing attention and neurofeedback effects, we tested whether time regulates alpha oscillations (H1.1–3; Fig 3).

#### Time up-/down-regulates alpha oscillations

First, we examined whether time up-/down-regulated alpha oscillations by comparing AAI values averaged within the first and second halves of each session. Specifically, we tested whether the averaged AAI would increase over time in the ALT_NF_ and decrease in ART_NF_ condition. The results provided moderate evidence that the AAI was higher in the second half of trials than in the first half of trials in the ART condition (BF_10_ = 6.22; Fig 3B). In the ALT condition, the results provided moderate evidence against a difference in the AAI between the first and the second half of trials (BF_10_ = 0.06; Fig 3B). Thus, time regulated alpha oscillations in one of the 2 conditions (ART), supporting hypothesis H1.1. Therefore, all subsequent analyses were based on averages across the second halves of trials.

#### Attention up-/down-regulates alpha oscillations

We next investigated whether attention up-/down-regulated alpha oscillations. We found strong evidence that the AAI, which reflects the relative alpha power over the right somatosensory regions compared to left somatosensory regions, was higher in the ART than in the ALT condition (BF_10_ = 51.90; Fig 3C). Thus, attention regulated alpha oscillations, supporting hypothesis H1.3.

#### Neurofeedback up-/down-regulates alpha oscillations

We further assessed whether neurofeedback enhanced the attention-related up-/down-regulation of alpha oscillations. The results provided strong evidence that the AAI was lower in the ALT verum than the ALT sham condition (BF_10_ = 25.71; Fig 3D). In the ART conditions, the results provided inconclusive evidence regarding an AAI difference between verum and sham conditions (BF_10_ = 1.02). Thus, neurofeedback regulated alpha oscillations in one of the 2 conditions (ALT), supporting hypothesis H1.2.

Taken together, the results correspond most closely to predicted result pattern 1 (Fig 1), which incorporates effects of time, attention, and neurofeedback on alpha oscillations.

### Pain perception was not up-/down-regulated by attention or neurofeedback

**Fig 4:**
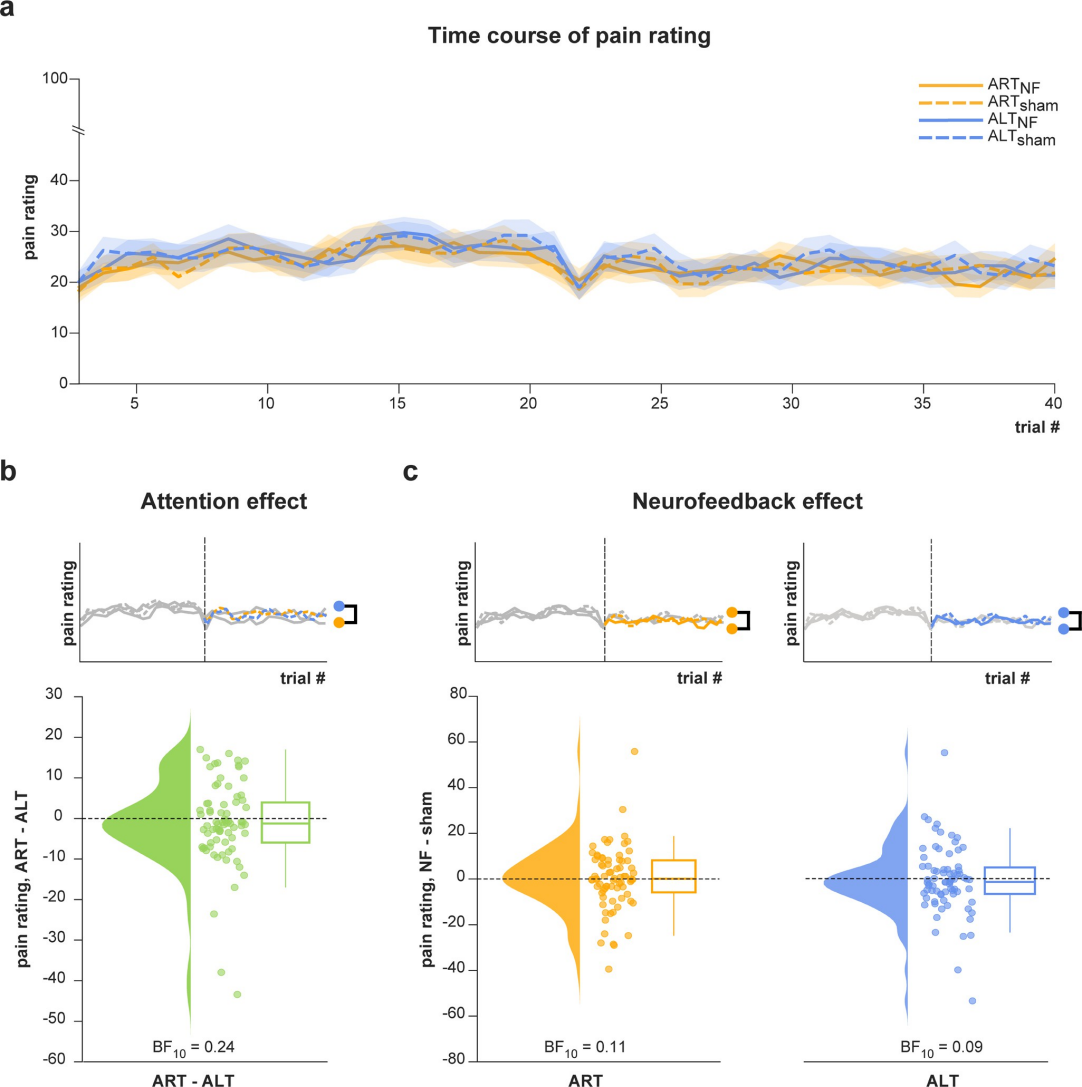
Observed result patterns characterizing attention and neurofeedback effects on pain perception. (a) Time course of the grand average pain rating over trials, separately for the verum neurofeedback (solid lines) and sham conditions (dashed lines) and the ART (yellow lines) and ALT conditions (blue lines), respectively. (b) Effect of attention on pain perception. The upper panel illustrates the analysis, which was performed on the second half of trials and included the contrast of the mean pain rating between the ART_sham_ and ALT_sham_ conditions, respectively. The lower panel shows the difference of single-participant mean pain ratings in the respective conditions. (c) Effect of neurofeedback on pain perception. The upper panel illustrates the analysis, which was performed on the second half of trials and included the contrast of the mean pain rating between the verum and sham conditions, calculated separately for the ART and ALT conditions. The lower panel shows the difference (verum—sham) of single-participant mean pain ratings for the ART (left) and ALT (right) conditions. Raincloud plots show un-mirrored violin plots displaying the probability density function of the data, individual data points, and boxplots. Boxplots depict the sample median, first (Q1), and third quartiles (Q3). Whiskers extend from Q1 to the smallest value within Q1–1.5* IQR and from Q3 to the largest values within Q3 + 1.5* IQR. The data underlying this figure can be found at https://osf.io/2mnys/. AAI, alpha asymmetry index; ALT, attention left training; ART, attention right training; BF, Bayes Factor; IQR, interquartile range; NF, neurofeedback.

Assuming that attention-related alpha oscillations are causally involved in the perception of pain, we expected pain ratings to be up- and down-regulated by attention and that these effects on pain perception would be enhanced through neurofeedback (H2.1–2; Fig 4).

#### Attention does not up-/down-regulate pain perception

We first investigated whether attention regulated pain perception. The results provided moderate evidence against pain ratings being lower during ART (i.e., when focusing on the non-stimulated hand) than ALT conditions (BF_10_ = 0.24; Fig 4B). Thus, although attention regulated alpha oscillations, it did not regulate pain perception, providing evidence against hypothesis H2.2.

#### Neurofeedback does not up-/down-regulate pain perception

We further assessed whether neurofeedback regulated pain perception. The results showed moderate evidence in the ART condition (BF_10_ = 0.11) and strong evidence in the ALT condition (BF_10_ = 0.09; Fig 4C) against a difference in pain ratings between verum and sham neurofeedback. Thus, although neurofeedback regulated alpha oscillations, it did not regulate pain perception, providing evidence against hypothesis H2.1.

### Brain responses were not up-/down-regulated by attention or neurofeedback

**Fig 5:**
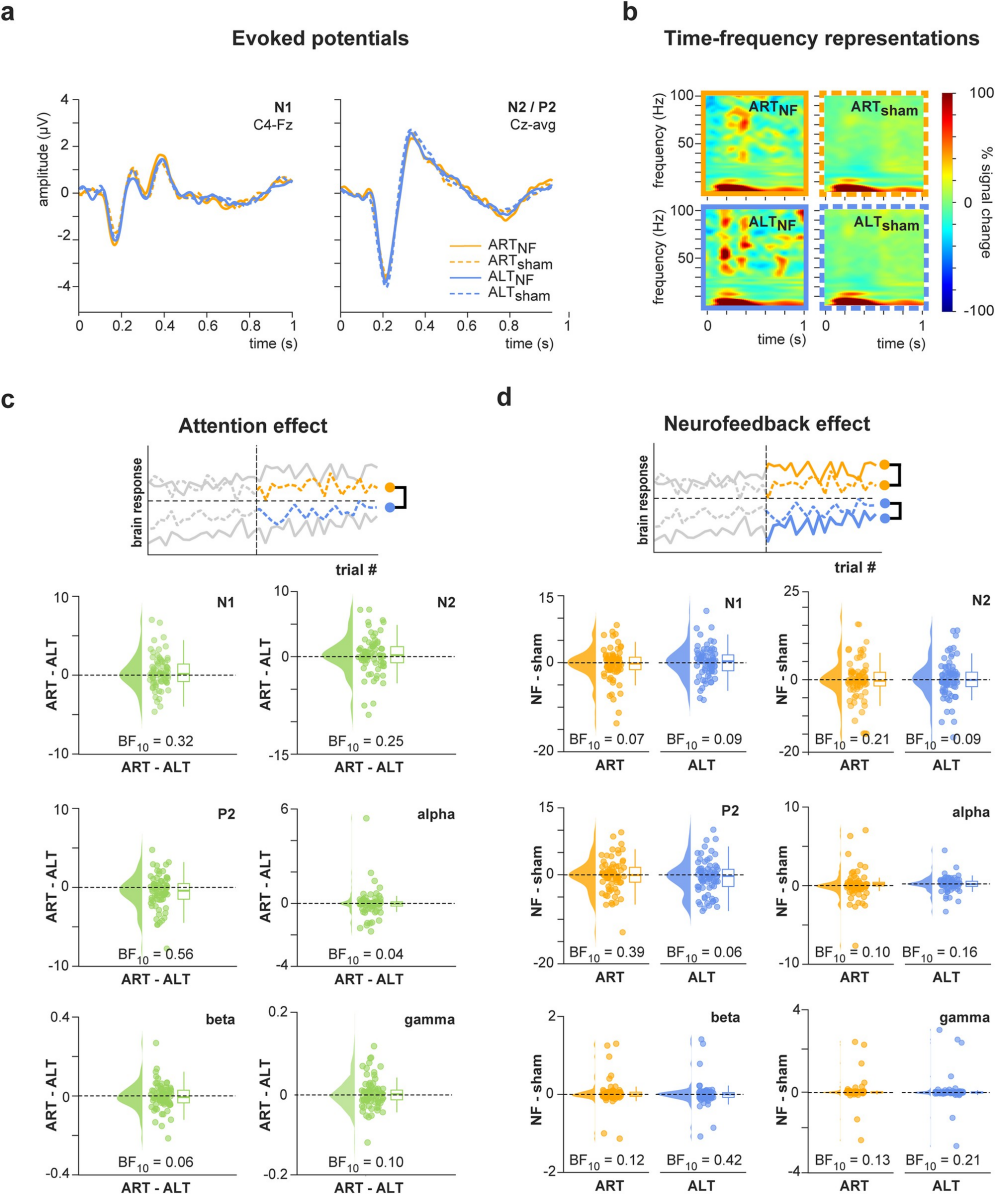
Observed result patterns characterizing attention and neurofeedback effects on pain-related brain responses. (a) Evoked potentials. The left panel shows the grand average time course of the N1 response for all conditions (verum/sham, ART/ALT), displayed at the contralateral central electrode C4, referenced to Fz. The right panel shows the grand average time course of the N2/P2 response for all conditions, displayed at the vertex electrode Cz, referenced to the average. (b) TFRs of neuronal activity recorded at electrode Cz in response to painful stimulation averaged across all subjects and per condition. For visualization only, TFRs are displayed as % signal change relative to a prestimulus baseline (−17 - −16 s). (c) Effect of attention on the N1, N2, and P2 response and alpha, beta, and gamma oscillations. The upper panel illustrates the analysis, which was performed on the second half of trials and included the contrast between the mean brain response in the ART_sham_ and ALT_sham_ conditions, respectively. The lower panel shows the difference (ART_sham_—ALT_sham_) of single-participant mean N1, N2, P2, alpha, beta, and gamma responses, respectively. (d) Effect of neurofeedback on the N1, N2, and P2 response and alpha, beta, and gamma oscillations. The upper panel illustrates the analysis, which was performed on the second half of trials and included the contrast between the mean brain response in the verum and sham conditions, calculated separately for the ART and ALT conditions. The lower panel shows the difference (verum–sham) of single-participant mean N1, N2, P2, alpha, beta, and gamma responses, respectively, for the ART (left) and ALT (right) conditions. Raincloud plots show un-mirrored violin plots displaying the probability density function of the data, individual data points, and boxplots. Boxplots depict the sample median, first (Q1), and third quartiles (Q3). Whiskers extend from Q1 to the smallest value within Q1–1.5* IQR and from Q3 to the largest values within Q3 + 1.5* IQR. The data underlying this figure can be found at https://osf.io/2mnys/. AAI, alpha asymmetry index; ALT, attention left training; ART, attention right training; BF, Bayes Factor; IQR, interquartile range; NF, neurofeedback; TFR, time–frequency representation.

We further investigated whether attention and neurofeedback up- or down-regulated brain responses to noxious stimuli (H2.3–4). To this end, we analyzed effects on the main components of the laser-evoked potential referred to as N1, N2, and P2 responses (Fig 5A) as well as on induced neuronal oscillations in the alpha, beta, and gamma frequency bands (Fig 5B).

#### Attention did not up-/down-regulate underlying brain responses

We first investigated whether attention regulated brain responses to painful stimuli. The results provided moderate to strong evidence against differences in brain responses between ALT and ART conditions (see Fig 5C for results including BFs). Thus, although attention regulated alpha oscillations, it did not regulate brain responses to pain, providing evidence against hypothesis H2.4.

#### Neurofeedback did not up-/down-regulate underlying brain responses

We further examined whether neurofeedback regulated brain responses to painful stimuli. The results showed moderate to strong evidence against differences in brain responses between neurofeedback and sham conditions (see Fig 5D for results including BFs). Thus, although neurofeedback regulated alpha oscillations, it did not regulate brain responses to pain, providing evidence against hypothesis H2.3.

### Alpha oscillations do not mediate attention effects on pain perception

Finally, we tested whether changes in attention, alpha oscillations, and pain perception can be integrated into a single, mechanistic model (H3). To this end, we performed a multilevel mediation analysis based on single-trial data. Specifically, we quantified to which extent alpha asymmetry mediates the relation between attentional focus and pain ratings.

**Fig 6:**
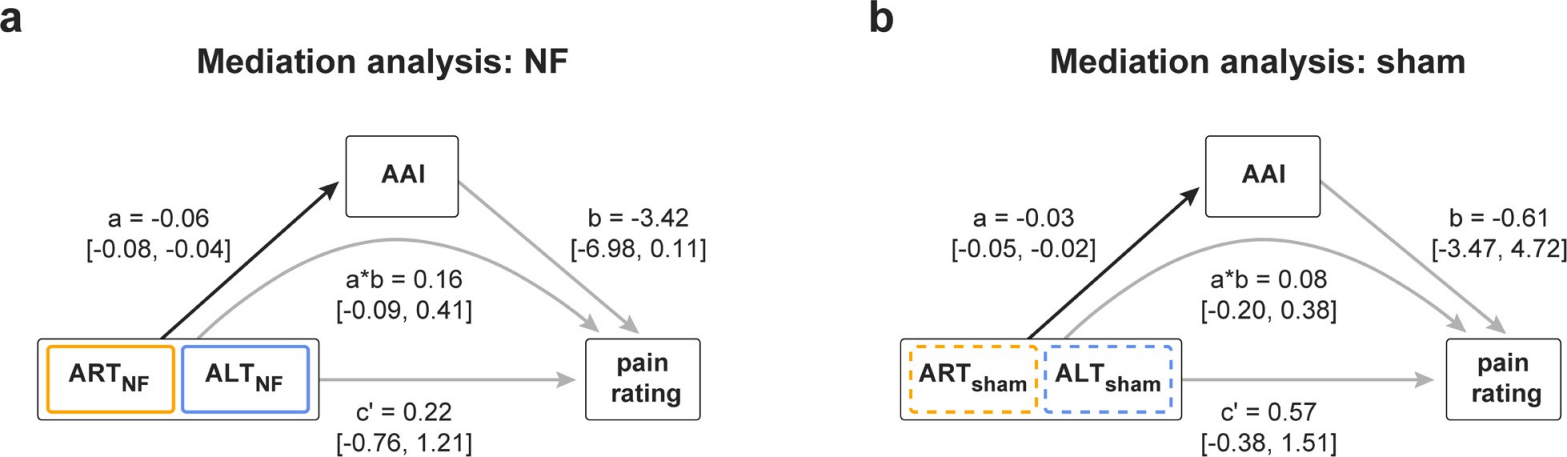
Mediation analysis. (a) Two-path mediation model in the verum condition. Path a represents the relationship between the attentional training condition (ART/ALT) and the AAI. Path b represents the relationship between the AAI and the pain rating, controlled for attentional training condition. Path c’ represents the relationship of attentional training condition to pain rating, controlled for AAI. Path a*b represents the mediation effect. The path coefficients and corresponding confidence intervals were calculated using Bayesian multilevel modeling and Markov Chain Monte Carlo procedures, both on a single-subject level (first-level coefficients) as well as on the group level (second-level coefficients). Depicted are second-level coefficients and corresponding confidence intervals only. Black arrows indicate a significant relationship/effect, gray arrows indicate a lack thereof. (b) Two-path mediation model in the sham condition. The data underlying this figure can be found at https://osf.io/2mnys/. AAI, alpha asymmetry index; ALT, attention left training; ART, attention right training; NF, neurofeedback.

For verum and sham neurofeedback (Fig 6), the results showed significant effects of condition (ART/ALT) on alpha asymmetry (path a, see figure for coefficients). However, the results did not show effects of alpha asymmetry (path b, see figure for coefficients) or condition (ART/ALT) on pain perception (path c’, see figure for coefficients). These findings are in line with the results of the pairwise comparisons reported above. Most importantly, the results did not show any evidence for a significant mediation effect (path a*b, see figure for coefficients).

Considering the lack of mediation effects, we did not perform the planned moderation analysis, which was designed to test for differences in the strength of mediation effects between verum and sham neurofeedback.

### Exploratory analyses

With 2 exploratory analyses we investigated (1) whether individual differences in learning could account for the null findings regarding pain modulation; and (2) whether the regulation of the AAI was sustained shortly before the application of the painful stimulus. The results align with the findings of the original analysis (see Supporting information).

## Discussion

To investigate the role of alpha oscillations in the processing and perception of pain, we performed a double-blind, sham-controlled EEG-based neurofeedback study. Our results provided strong evidence that attention and neurofeedback modulate alpha oscillations over somatosensory regions. However, we did not find evidence that alpha oscillations shape the perception of pain. Specifically, we found evidence against an influence of attention and neurofeedback on pain perception and pain-related brain responses. This dissociation of effects on alpha oscillations and pain challenges the hypothesis of a causal link between alpha oscillations and pain.

The successful regulation of alpha asymmetry by modulations of spatial attention and neurofeedback aligns with our hypotheses. Moreover, our study confirms that neurofeedback effects on alpha oscillations can be achieved even within a single, relatively short (<30 min) training session [30,31,40]. Short-term neurofeedback protocols are still relatively rare, and most traditional protocols include 5 or more sessions [69,70]. As the present study adhered to recent recommendations for neurofeedback studies [17] and was conducted as a Registered Report, it delivers high-quality evidence for short-term, attention-based neurofeedback being effective in modulating brain activity. These findings hold significant implications for future neurofeedback studies, particularly regarding the refinement and optimization of short-term protocols. Such advancements are pivotal for making neurofeedback a viable, practical, and accessible treatment option.

Our results provided evidence against attention effects on pain perception. This lack of attention effects on pain, which have been shown in numerous previous studies [71,72], can be explained in different ways. First, participants might not have focused their attention on the left or right hand as instructed. However, this appears unlikely as attention showed strong effects on alpha asymmetry. Second, the neurofeedback task, being both highly demanding and rewarding, might have distracted participants from the painful stimuli and, thereby, overshadowed any attention effects on pain perception. This possibility appears plausible, particularly given the rather low saliency of the repeatedly applied and predictable painful stimuli.

Further, our results provided evidence against neurofeedback effects on pain perception while previous evidence on neurofeedback effects on pain perception has been inconsistent [20,28]. The present findings extend the previous evidence by following recent recommendations for performing neurofeedback studies [17], including an adequate (sham) control condition and evaluating brain activity and pain perception during neurofeedback. Moreover, we relied on the rigorous Registered Report format, which reduces reporting and publication biases [73]. However, our findings do not rule out that neurofeedback can successfully modulate pain perception using other training protocols, neurofeedback targets, and types of pain.

We observed that attention and neurofeedback modulated alpha oscillations but not the processing and perception of pain. This dissociation indicates that a causal relationship between alpha oscillations and pain (1) might not exist; (2) might demand stronger modulations of alpha oscillations; or (3) might be more complex than expected. First, a lack of a causal relationship appears possible. Previous studies reporting a relationship between somatosensory alpha oscillations and pain perception were based on correlations between passively observed brain activity and perception ([3–7,74,75]; for review see [16]). However, these correlations allow for weaker causal inferences than assessing the effects of actively modulating brain activity, as done in the present study. Thus, a fundamental lack of a causal relationship between alpha oscillations and pain perception cannot be ruled out. Second, although we observed strong evidence for neurofeedback-induced modulations of somatosensory alpha oscillations, the magnitude of these modulations may not have been sufficient to produce measurable behavioral changes, i.e., modulations of pain perception. Lastly, the results might indicate that the relationship between alpha oscillations and pain is more complex than expected. Pain is a complex sensory, affective, and cognitive experience driven by sensory and contextual information. Correspondingly, the cerebral processing of pain involves complex patterns of brain activity and connectivity at different frequencies and locations [1,2,76,77]. Thus, alpha oscillations might play a causal role in pain perception only in conjunction with certain contextual factors and other neural features. Such a multi-causal relationship might be considered in future studies.

Finally, our study did not demonstrate a modulation of pain perception by attention-based neurofeedback nor a mechanistic link between attention, alpha oscillations, and pain perception. Thus, it remains unclear whether somatosensory alpha oscillations represent an appropriate target for noninvasive brain modulation techniques. However, considering the evidence for a role of alpha oscillations as a sensory gating mechanism in other modalities [40,78,79], we think that investigating its effects on pain perception remains a promising direction for future research. Future studies might not only use neurofeedback to modulate alpha oscillations and pain, but can use other strategies to modulate oscillations such as transcranial alternating current stimulation (tACS, [80]) or sensory entrainment [81].

Taken together, our study demonstrates that somatosensory alpha oscillations can be successfully modulated by short-term (<30 min) attention-based neurofeedback. This indicates the potential of noninvasive neuromodulatory techniques even within short-term designs. However, these modulations do not necessarily result in modulations of pain perception and pain processing. This challenges the hypothesis of a direct causal link between alpha oscillations and pain perception. Thus, adhering to rigorous standards of open and reproducible science, our study prompts a further exploration of the relationship between alpha oscillations and pain.

## Supporting information

S1 Text Fig A in S1 Text. **Bayes factor design analysis**. Performance of a sequential Bayes factor design with Nmax = 95 under H0 (left) and H1 (right). **Fig B in S1 Text**. **Performance of a sequential Bayes factor design with different effect size estimates and sample sizes**. Graphs depict the percentage of simulated studies (10,000) terminating at the H1 boundary (BF10 ≥ 10, purple diamonds) and the H0 boundary (BF10 ≤ 1/10, orange circles) for the simulated effect sizes Cohen’s d = 0.23, d = 0.41, d = 0.53, d = 1.31 and the sample sizes *N* = 20, *N* = 40, *N* = 60, *N* = 80, and *N* = 100. Chosen effect size estimates were derived based on the following assumptions: (1) Cohen’s d = 0.23: sample with 30% non-responders with an ES of d = 0.2 and 70% responders with an ES of d = 0.3; (2) Cohen’s d = 0.41: sample with 30% non-responders with an ES of d = 0.2 and 70% responders with an ES of d = 0.5, (3) Cohen’s d = 0.53: sample with 30% non-responders with an ES of d = 0.2 and 70% responders with an ES of d = 0.7, and (4) Cohen’s d = 1.31 based on effect sizes from previous related work. BF, Bayes factor; ES, effect size estimate. **Table A in S1 Text. Overview of effect sizes reported in short-term neurofeedback studies focusing on the modulation of alpha oscillations. Fig C in S1 Text. Stimulus material and real-time data analysis**. (a) Neutral face images selected from the Averaged Karolinska Directed Emotional Faces data set [2] (image IDs from left to right: FNEFL, FNEHL, FNEFR, FNEHR, MNEFL, MNEHL, MNEFR, and MNEHR). To support image fixation, a small, central fixation cross is superimposed on the images during verum and sham conditions. (b) Every 100 ms, 1,000 ms data segments are extracted from the buffer and alpha power for left and right somatosensory regions is calculated. Subsequently, power values are used to calculate the AAI for each segment. To avoid sudden jumps in the feedback signal, a weighting function is applied to the AAI time course before visualization. Specifically, AAI values within a time-window of 3,000 ms are weighted with a linear weighting function. The resulting AAI value is then used to determine the visibility parameter c which controls the visibility of the selected face image. The face stimulus shown as an example for the visual feedback is derived from [2], image ID: FNES. **Fig D in S1 Text. AAI analysis pipeline.** AAI values were analyzed using an adaptive analysis pipeline focusing on time, neurofeedback, and attention effects. Decisions regarding data selection (e.g., usage of data_2ndhalf vs. data_total) were saved as indicated by the cylinder-shaped data storage symbol. AAI, alpha asymmetry index; ALT, attention left training; ART, attention right training. **Fig E in S1 Text. Pain analysis pipeline.** Pain ratings were analyzed by repeating the AAI analysis steps including the decisions on data selection. Information regarding these decisions were retrieved as indicated by the cylinder-shaped data storage symbol. Diverging from AAI analyses, the direction of pairwise comparisons was reversed, however, and time effects were not be evaluated. This procedure was also used for pain-related evoked and oscillatory brain responses. AAI, alpha asymmetry index; ALT, attention left training; ART, attention right training; DV, dependent variable. **Fig F in S1 Text. Quantification of evoked and oscillatory brain responses to noxious stimuli.** Validation of the proposed procedures to determine (a) evoked responses and (b) oscillatory brain responses to noxious stimuli based on a published data set [3] (*n* = 48, 20 trials) using the same means of noxious stimulation (Deka Stimul 1340 stimulator, stimulus duration: 4 ms, stimulus diameter: 7 mm, stimulus intensity: 3.5 J). Mean time courses and TFRs averaged across trials and participants are shown. Marked time points in (a) represent averaged peak latencies which were used to quantify N1, N2, and P2 responses and shadings indicate the standard error of the mean. Marked time-frequency windows in (b) indicate windows chosen to quantify alpha, beta, and gamma responses. Topographies depict the scalp distribution of neural activity for these time points/time-frequency windows. For visualization purposes, the TFR is displayed as %-signal change relative to a prestimulus baseline (−3.3 to −2.8 s). TFR, time-frequency representation. **Fig G in S1 Text. AAI modulation through neurofeedback training.** Results of a pilot study with *n* = 5 participants completing 36 trials of ARTNF and ALTNF training. Plots show (a) mean group effects with shadings indicating the SEM as well as (b) individual data. The neurofeedback training protocol was identical to the one described above but entailed longer trial durations (15, 20, or 25 s) and no laser stimuli. AAI, alpha asymmetry index; ALTNF, verum attention left training; ARTNF, verum attention right training; SEM, standard error of the mean. **Fig H in S1 Text**. **CONSORT flow diagram of study recruitment.**
(PDF)

## Abbreviations

AAI: alpha asymmetry index
AKDEF: Averaged Karolinska Directed Emotional Faces
AI: artefact index
ALT: attention left training
ART: attention right training
BF: Bayes factor
BFDA: Bayes factor design analysis
BI: blinding indices
BR: brain response
CONSORT: Consolidated Standards of Reporting Trials
EEG: electroencephalography
GSE: General Self-Efficacy Scale
ICA: independent component analysis
IQR: interquartile range
M: mediator
MHLC: Multidimensional Health Locus of Control
NF: neurofeedback
NRS: numerical rating scale
PANAS: Positive and Negative Affect Schedule
SBF: sequential Bayes factor
tACS: transcranial alternating current stimulation
TFR: time-frequency representation
VAS: visual analogue scale
X: indepedent variable
Y: dependent variable
