# Supplementary material for: Neurofeedback and attention modulate somatosensory alpha oscillations but not pain perception
Source: PLoS Biol. 2025 Jan 23;23(1):e3002972. doi: 10.1371/journal.pbio.3002972 (PMC11756787; doi:10.1371/journal.pbio.3002972)
Supplement: S1 Text — Bayes factor design analysis. Performance of a sequential Bayes factor design with Nmax = 95 under H0 (left) and H1 (right). Fig B in S1 Text. Performance of a sequential Bayes factor design with different effect size estimates and sample sizes. Graphs depict the percentage of simulated studies (10,000) terminating at the H1 boundary (BF10 ≥ 10, purple diamonds) and the H0 boundary (BF10 ≤ 1/10, orange circles) for the simulated effect sizes Cohen’s d = 0.23, d = 0.41, d = 0.53, d = 1.31 and the sample sizes N = 20, N = 40, N = 60, N = 80, and N = 100. Chosen effect size estimates were derived based on the following assumptions: (1) Cohen’s d = 0.23: sample with 30% non-responders with an ES of d = 0.2 and 70% responders with an ES of d = 0.3; (2) Cohen’s d = 0.41: sample with 30% non-responders with an ES of d = 0.2 and 70% responders with an ES of d = 0.5, (3) Cohen’s d = 0.53: sample with 30% non-responders with an ES of d = 0.2 and 70% responders with an ES of d = 0.7, and (4) Cohen’s d = 1.31 based on effect sizes from previous related work. BF, Bayes factor; ES, effect size estimate. Table A in S1 Text. Overview of effect sizes reported in short-term neurofeedback studies focusing on the modulation of alpha oscillations. Fig C in S1 Text. Stimulus material and real-time data analysis. (a) Neutral face images selected from the Averaged Karolinska Directed Emotional Faces data set [2] (image IDs from left to right: FNEFL, FNEHL, FNEFR, FNEHR, MNEFL, MNEHL, MNEFR, and MNEHR). To support image fixation, a small, central fixation cross is superimposed on the images during verum and sham conditions. (b) Every 100 ms, 1,000 ms data segments are extracted from the buffer and alpha power for left and right somatosensory regions is calculated. Subsequently, power values are used to calculate the AAI for each segment. To avoid sudden jumps in the feedback signal, a weighting function is applied to the AAI time course before visualization. Specifically, AAI values within [file pbio.3002972.s001.pdf]

Supplementary Material

Neurofeedback and attention modulate somatosensory alpha oscillations but not pain perception

Vanessa D. Hohn[1,2,co], Laura Tiemann[1,2,co], Felix S. Bott[1,2,co], Elisabeth S. May[1,2], Clara Fritzen[1,2], Moritz M. Nickel[1,2], Cristina Gil Ávila[1,2], and Markus Ploner[1,2,3] *

[1] Department of Neurology, School of Medicine and Health, Technical University of Munich (TUM), Munich, Germany

[2] TUM-Neuroimaging Center, School of Medicine and Health, TUM, Munich, Germany

[3] Center for Interdisciplinary Pain Medicine, School of Medicine and Health, TUM, Munich, Germany

[co] contributed equally to this work

* markus.ploner@tum.de

Bayes factor design analysis

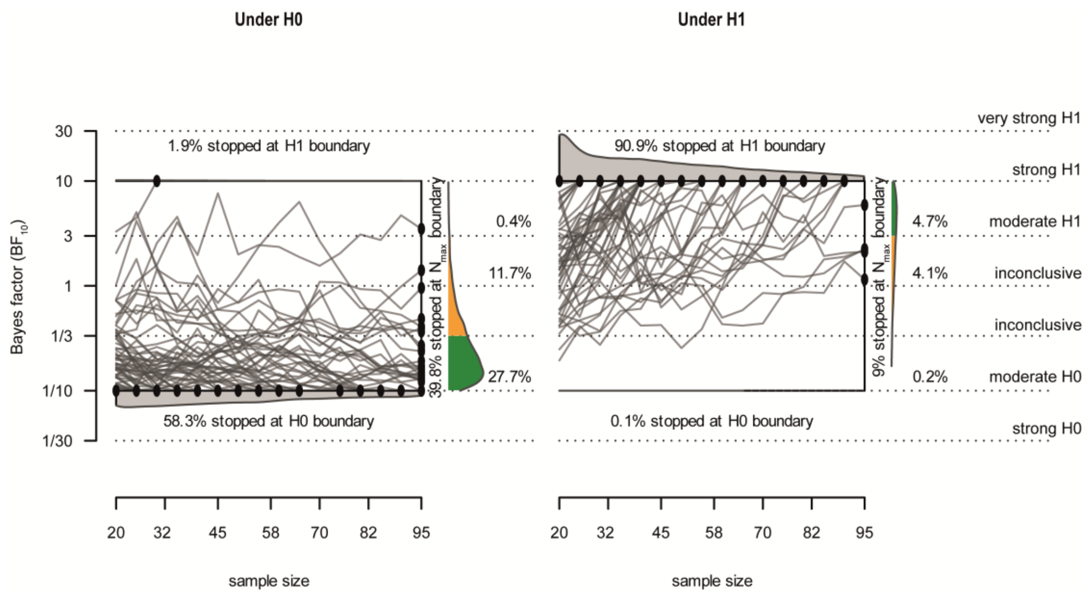

Fig A. Bayes factor design analysis. Performance of a sequential Bayes factor design with Nmax = 95 under H0 (left) and H1 (right).

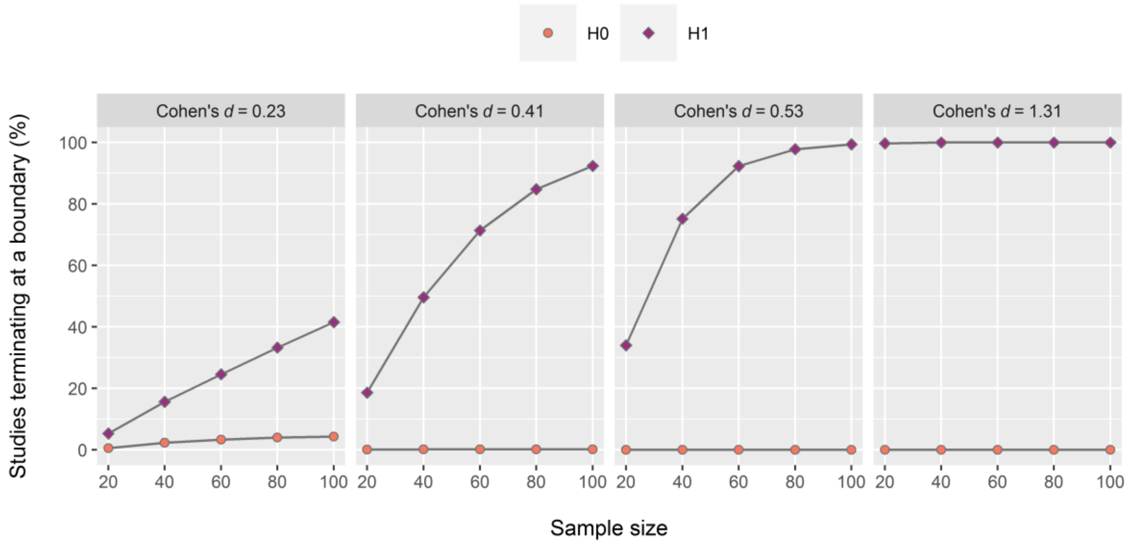

Fig B. Performance of a sequential Bayes factor design with different effect size estimates and sample sizes. Graphs depict the percentage of simulated studies (10000) terminating at the H1 boundary (BF10 ≥ 10, purple diamonds) and the H0 boundary (BF10 ≤ 1/10, orange circles) for the simulated effect sizes Cohen's d = 0.23, d = 0.41, d = 0.53, d = 1.31 and the sample sizes N = 20, N = 40, N = 60, N = 80, and N = 100. Chosen effect size estimates were derived based on the following assumptions: (1) Cohen's d = 0.23: sample with 30% non-responders with an ES of d = 0.2 and 70% responders with an ES of d = 0.3; (2) Cohen's d = 0.41: sample with 30% non-responders with an ES of d = 0.2 and 70% responders with an ES of d = 0.5, (3) Cohen's d = 0.53: sample with 30% non-responders with an ES of d = 0.2 and 70% responders with an ES of d = 0.7, and (4) Cohen's d = 1.31 based on effect sizes from previous related work. BF, Bayes factor; ES, effect size estimate.

Table A. Overview of effect sizes reported in short-term neurofeedback studies focusing on the modulation of alpha oscillations

	**Okazaki 2015**	**Brickwedde 2019**	**Bagherzadeh 2020**
paradigm	bi-directional regulation between-subjects design with four groups (n = 10 NFB left alpha asymmetry, n = 7 NFB right alpha asymmetry, n = 10 sham left, n = 9 sham right), single session, MEG study	bi-directional regulation between-subjects design with three groups (n = 17 NFB alpha up, n = 15 NFB alpha down, n = 20 control), two sessions, EEG study	bi-directional regulation between-subjects design with two groups (n = 10 NFB left alpha asymmetry, n = 10 NFB right alpha asymmetry), single session, MEG study
statistics	two-way ANOVAs, focus on post hoc one-way ANOVAs comparing alpha asymmetry between NFB groups	two-way ANOVAs, focus on post hoc t-tests comparing alpha power between NFB groups	two-sided permutation test comparing left vs right alpha power separately for both NFB groups<br>*Note:* No direct comparison between groups was performed. Due to significant asymmetry in both conditions, direct comparisons between both groups are likely to yield even larger effect sizes.
source	manuscript page 5	supplement:<br>table 1 and 2: neuronal data session 1<br>table 3: neuronal data session 2	manuscript page 580

	Okazaki 2015	Brickwedde 2019	Bagherzadeh 2020
results	- NFB left alpha asymmetry vs NFB right alpha asymmetry group: *F*(1, 31) = 12.34, *p* = 0.01, Cohen's d = 1.843<br><br>Effect size: Cohen's d = 1.84	- table 1: alpha up vs alpha down group (raw power)<br>- block 1: not reported<br>- block 2: not reported<br>- block 3: *t*(30) = 2.00, p = 0.055, Cohen's d = 0.708<br>- table 2: Alpha up vs alpha down group (normalized power)<br>- block 1: *t*(30) = 2.46, p = 0.020, Cohen's d = 0.871<br>- block 2: *t*(30) = 3.95, p = 0.000, Cohen's d = 1.399<br>- block 3: *t*(30) = 3.71, p = 0.001, Cohen's d = 1.314<br>- table 3: Alpha up vs alpha down group (normalized power)<br>- block 1: *t*(30) = 3.39, p = 0.002, Cohen's d = 1.201<br>- block 2: *t*(30) = 3.83, p = 0.001, Cohen's d = 1.357<br><br>Mean effect size: Cohen's d = 1.14	- left vs right alpha power (NFB left alpha asymmetry group)<br>- first block: *p* = 0.066, Cohen's d = 0.70<br>- last block: *p* = 0.002, Cohen's d = 1.45<br>- left vs right alpha power (NFB right alpha asymmetry group)<br>- first block: *p* = 0.590, Cohen's d = 0.19<br>- last block: *p* = 0.008, Cohen's d = 1.44<br><br>Mean effect size: Cohen's d = 0.95

Note. Effect sizes were calculated using the online resource psychometrica [1] in consultation with a statistician. During data extraction, we focused on active, verum neurofeedback conditions available in each study for consistency. In addition, we focused on post hoc pairwise comparisons because (1) these are closer to the analyses proposed in the current study and because (2) not all manuscripts contain sufficient information to calculate effect size estimates for the reported interaction effects [1].

Image visibility

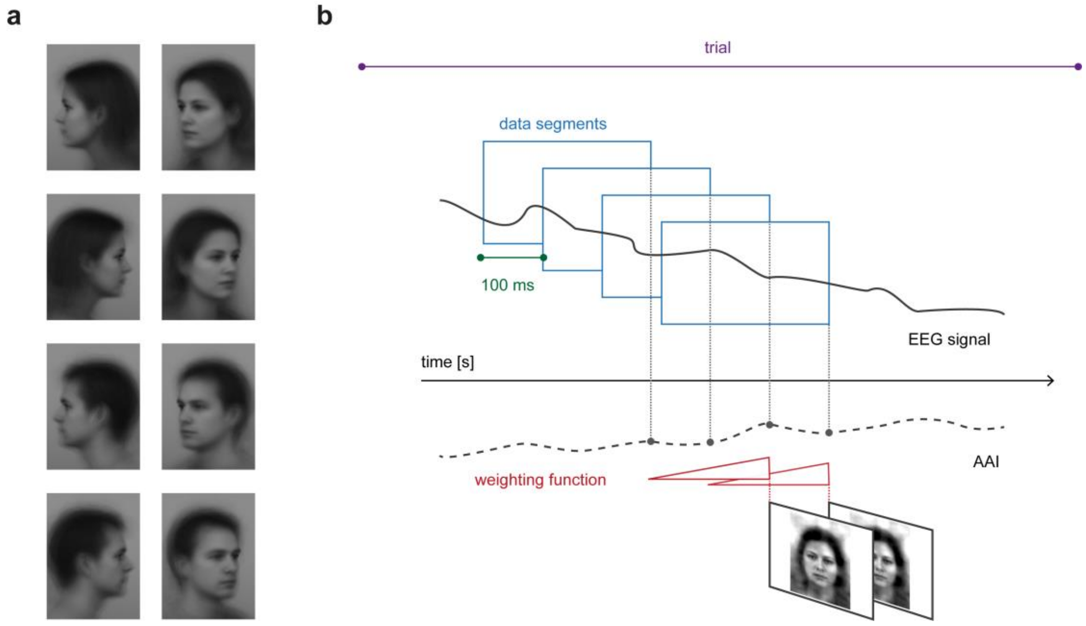

Fig C. Stimulus material and real-time data analysis. (a) Neutral face images selected from the Averaged Karolinska Directed Emotional Faces data set [2] (image IDs from left to right: FNEFL, FNEHL, FNEFR, FNEHR, MNEFL, MNEHL, MNEFR, MNEHR). To support image fixation, a small, central fixation cross is superimposed on the images during verum and sham conditions. (b) Every 100 ms, 1000 ms data segments are extracted from the buffer and alpha power for left and right somatosensory regions is calculated. Subsequently, power values are used to calculate the AAI for each segment. To avoid sudden jumps in the feedback signal, a weighting function is applied to the AAI time course before visualization. Specifically, AAI values within a time-window of 3000 ms are weighted with a linear weighting function. The resulting AAI value is then used to determine the visibility parameter c which controls the visibility of the selected face image. The face stimulus shown as an example for the visual feedback is derived from [2], image ID: FNES.

Analysis pipelines

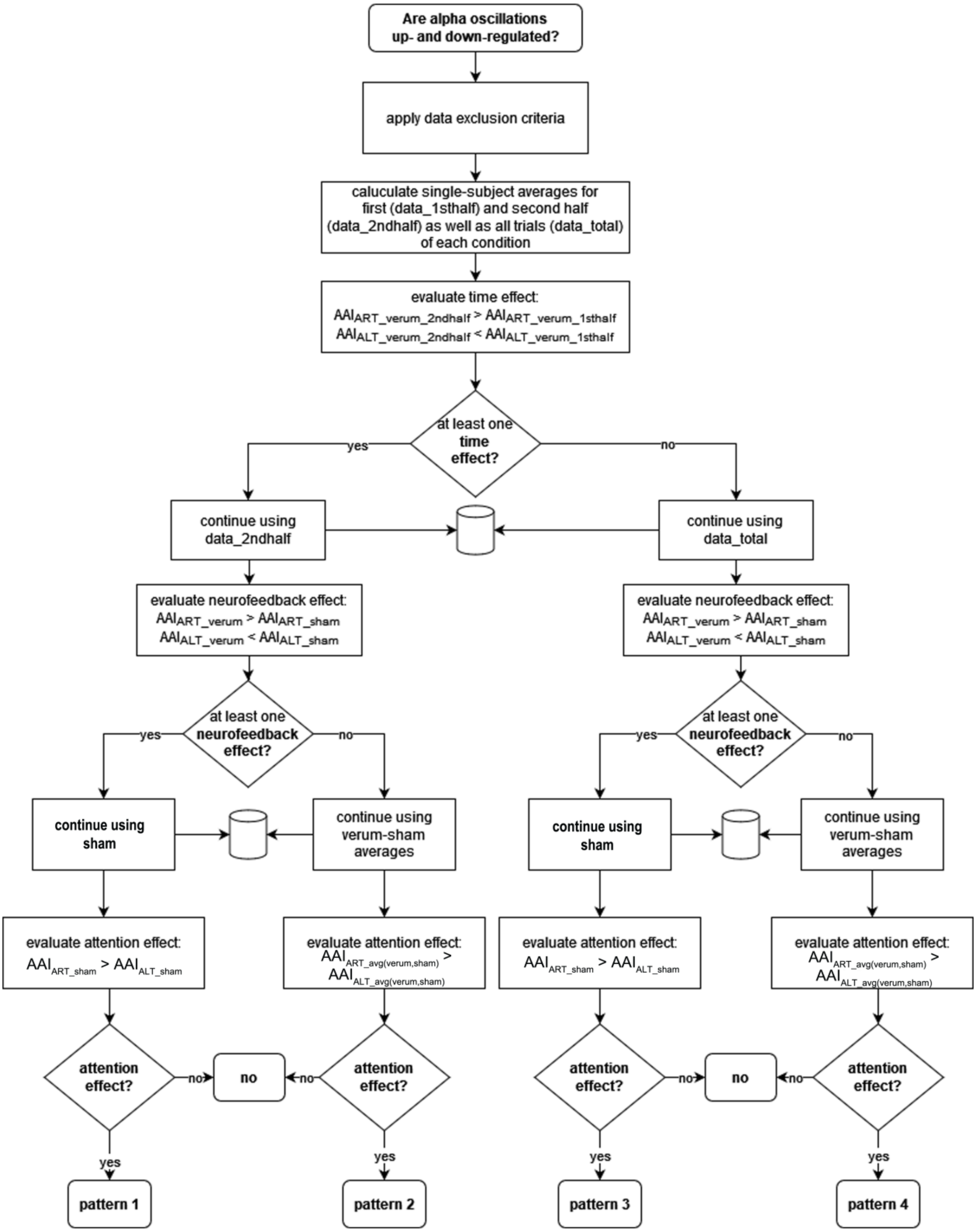

Fig D. AAI analysis pipeline. AAI values were analyzed using an adaptive analysis pipeline focusing on time, neurofeedback, and attention effects. Decisions regarding data selection (e.g., usage of data_2ndhalf vs data_total) were saved as indicated by the cylinder-shaped data storage symbol. AAI, alpha asymmetry index; ALT, attention left training; ART, attention right training.

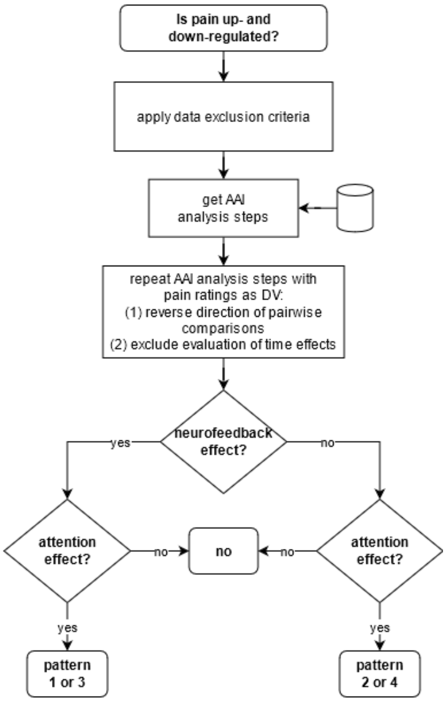

Fig E. Pain analysis pipeline. Pain ratings were analyzed by repeating the AAI analysis steps including the decisions on data selection. Information regarding these decisions were retrieved as indicated by the cylinder-shaped data storage symbol. Diverging from AAI analyses, the direction of pairwise comparisons was reversed, however, and time effects were not be evaluated. This procedure was also used for pain-related evoked and oscillatory brain responses. AAI, alpha asymmetry index; ALT, attention left training; ART, attention right training; DV, dependent variable.

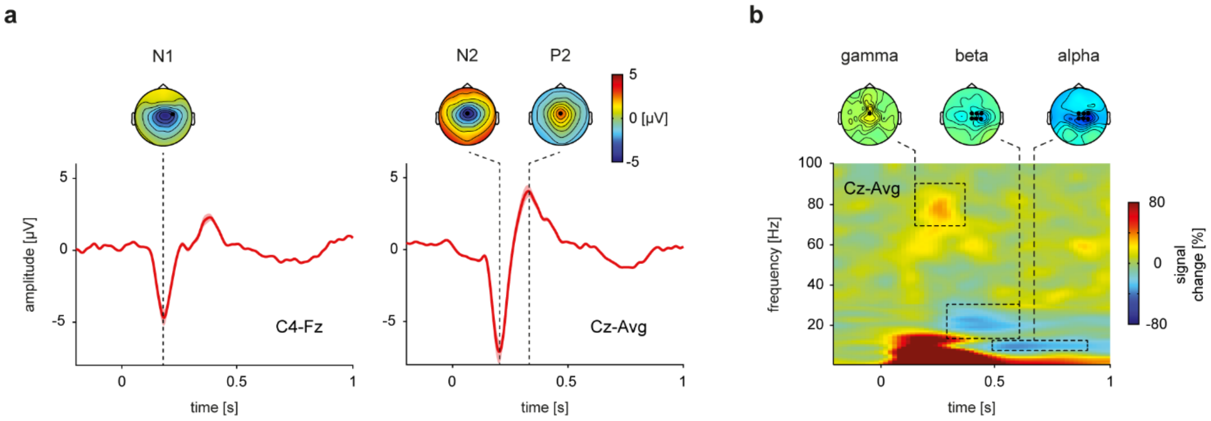

Fig F. Quantification of evoked and oscillatory brain responses to noxious stimuli. Validation of the proposed procedures to determine (a) evoked responses and (b) oscillatory brain responses to noxious stimuli based on a published data set[3] (n = 48, 20 trials) using the same means of noxious stimulation (Deka Stimul 1340 stimulator, stimulus duration: 4 ms, stimulus diameter: 7 mm, stimulus intensity: 3.5 J). Mean time courses and TFRs averaged across trials and participants are shown. Marked time points in (a) represent averaged peak latencies which were used to quantify N1, N2, and P2 responses and shadings indicate the standard error of the mean. Marked time-frequency windows in (b) indicate windows chosen to quantify alpha, beta, and gamma responses. Topographies depict the scalp distribution of neural activity for these time points/time-frequency windows. For visualization purposes, the TFR is displayed as %-signal change relative to a prestimulus baseline (-3.3 to -2.8 s). TFR, time-frequency representation.

Pilot data

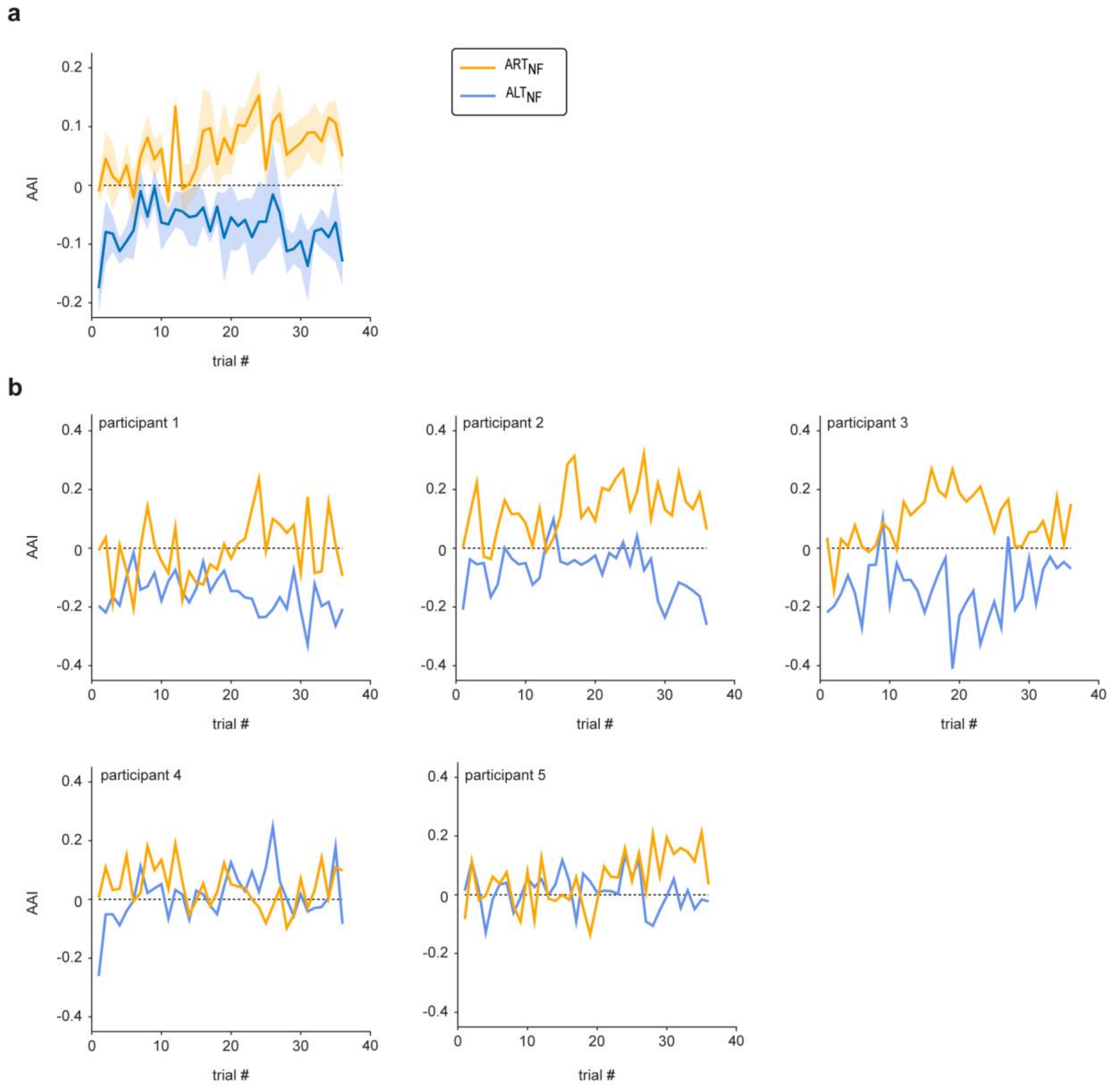

Fig G. AAI modulation through neurofeedback training. Results of a pilot study with n = 5 participants completing 36 trials of ART$_{NF}$ and ALT$_{NF}$ training. Plots show (a) mean group effects with shadings indicating the SEM as well as (b) individual data. The neurofeedback training protocol was identical to the one described above but entailed longer trial durations (15, 20, or 25 s) and no laser stimuli. AAI, alpha asymmetry index; ALT$_{NF}$, verum attention left training; ART$_{NF}$, verum attention right training; SEM, standard error of the mean.

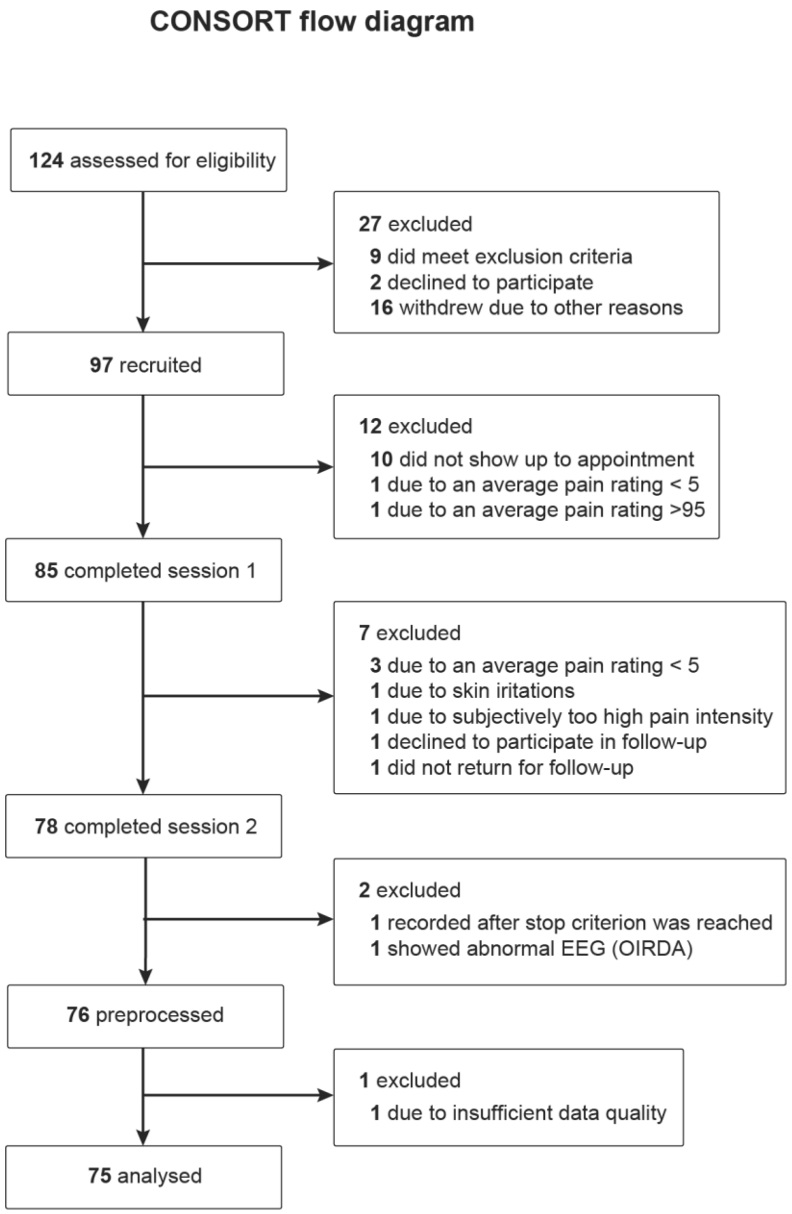

Fig H. **CONSORT flow diagram of study recruitment**.

Exploratory analysis

Attention does not up/downregulate pain perception in a subgroup of successful regulators. About two-thirds of participants (n = 47, 62.7%; Fig 3C) successfully achieved modulation of somatosensory alpha oscillations in the targeted direction. To investigate whether individual differences in learning could account for the null findings regarding pain modulation, we repeated the main analysis (H1) for this subgroup of successful regulators. The results showed inconclusive evidence regarding an attention effect on pain in terms of lower pain ratings during the ART (i.e., focusing on the non-stimulated hand) than the ALT conditions (BF10 = 0.6). Thus, even among participants who successfully regulated alpha oscillations, we found no evidence for an effect on pain perception.

Modulation of alpha oscillations was sustained during prestimulus phase. To address the question of whether the regulation of the AAI was sustained shortly before the painful stimulus, we repeated the primary analysis (H1) for the last second before stimulus application.

Following the original analysis, we first examined whether time up/downregulated alpha oscillations by comparing AAI values averaged within the first and second halves of each session. The results provided moderate evidence against a difference between the two halves in both ART and ALT conditions (BF10 = 0.11). Based on this finding, we analyzed the entire set of trials to assess attentional and neurofeedback effects on AAI.

Next, we investigated whether attention up/downregulated alpha oscillations. We found strong evidence that the AAI was higher in the ART than in the ALT condition during the final second before stimulation (BF_{10} = 16.88; original analysis: BF_{10} = 51.90). This suggests that attention-related alpha modulation was indeed sustained in the prestimulus phase.

We further assessed whether neurofeedback enhanced the attention-effect by comparing verum and sham conditions. Consistent with the results of the original analysis, we found strong evidence that the AAI was lower in the ALT verum than in the ALT sham condition (BF_{10} = 8.26; original analysis: BF_{10} = 25.71). For the ART condition, evidence pointed against a difference between verum and sham conditions (BF_{10} = 0.31; original analysis: inconclusive evidence, BF_{10} = 1.02). Thus, as in the original analysis, neurofeedback regulated alpha oscillations in one of the two conditions (ALT).

Together, these findings confirm that the impact of attention and neurofeedback on alpha modulation persisted through the final second before stimulus application, making it unlikely that the null findings in pain perception result from a lack of alpha modulation in this critical prestimulus phase.

References

1. Lenhard W, Lenhard A. Computation of effect sizes 2016 [cited 2022 011.06.2022]. Available from: https://www.psychometrica.de/effect_size.html.
2. Lundqvist D, Litton JE. The averaged Karolinska directed emotional faces - AKDEF. Stockholm: Department of Clinical Neuroscience, Psychology section, Karolinska Institutet; 1998. p. The averaged Karolinska directed emotional faces - AKDEF.
3. Nickel MM, Tiemann L, Hohn VD, May ES, Gil Avila C, Eippert F, Ploner M. Temporal-spectral signaling of sensory information and expectations in the cerebral processing of pain. Proc Natl Acad Sci U S A. 2022;119(1). doi: 10.1073/pnas.2116616119. PubMed PMID: 34983852; PubMed Central PMCID: PMCPMC8740684.
